# Patient-specific functional brain architecture explains cortical patterns of tau PET in Alzheimer’s disease

**DOI:** 10.1101/2025.10.02.679969

**Published:** 2025-10-14

**Authors:** Harry H. Behjat, Jacob W. Vogel, Olof Strandberg, Nicola Spotorno, Jonathan Rittmo, Sara Stampacchia, Lyduine E. Collij, Alexa Pichet Binette, Yu Xiao, Pavanjit Chaggar, Danielle van Westen, Erik Stomrud, Sebastian Palmqvist, Niklas Mattsson-Carlgren, Dimitri Van De Ville, Ruben Smith, Oskar Hansson, Rik Ossenkoppele

**Affiliations:** 1 Clinical Memory Research Unit, Department of Clinical Sciences Malmö, Faculty of Medicine, Lund University, Lund, Sweden; 2 Neuro-X Institute, École Polytechnique Fédérale de Lausanne, Geneva, Switzerland; 3 Neurodegenerative Research Unit, Department of Clinical Sciences Malmö, Faculty of Medicine, SciLifeLab, LU, Lund, Sweden; 4 Radiology and Nuclear Medicine, Amsterdam UMC, location VUmc, Amsterdam, the Netherlands; 5 Brain Imaging, Amsterdam Neuroscience, Amsterdam, the Netherlands; 6 Department of Physiology and Pharmacology, Université de Montréal, Montréal, Quebec, Canada; 7 Centre de Recherche de l’Institut Universitaire de Gériatrie de Montréal, Montréal, Quebec, Canada; 8 Department of Diagnostic Radiology, Department of Clinical Sciences Lund, Lund University, Lund, Sweden; 9 Memory Clinic, Skåne University Hospital, SE-205 02 Malmö, Sweden; 10 Department of Radiology and Medical Informatics, University of Geneva, Geneva, Switzerland; 11 Alzheimer Center Amsterdam, Neurology, Vrije Universiteit Amsterdam, Amsterdam UMC, Amsterdam, the Netherlands; 12 Amsterdam Neuroscience, Neurodegeneration, Amsterdam, the Netherlands; 13 These authors jointly supervised this work.

## Abstract

The spatial distribution of tau pathology, the core driver of neurodegeneration in Alzheimer’s disease (AD), varies markedly across individuals. While tau is thought to spread along brain networks, the role of inter-individual variability in shaping these patterns remains underexplored. Using resting-state fMRI and tau-PET from 805 participants across the AD continuum, we studied whether subject-specific functional connectivity (FC) profiles enhance the characterization of tau deposition patterns. A hybrid approach integrating individual and group-average FC outperformed both alone, particularly in symptomatic individuals and at finer spatial resolutions, the latter underscoring a critical but often overlooked role of spatial scale. Individualized FC also better captured individual tau topographies than canonical tau-PET maps derived from cohort-level data. These effects were specific to tau, and not seen for β-amyloid, and their predictive power increased with spatial granularity. Furthermore, baseline FC also predicted future tau accumulation at the individual level, supporting its prognostic value. Together, these findings provide strong evidence that individual functional brain architecture shapes tau propagation in humans, supporting the network spread hypothesis by showing that variability in connectivity translates into heterogeneity in tau distribution. This work advances biological understanding of tau propagation in AD, highlighting functional connectivity as a mechanistic substrate that supports prognostic assessment of tau trajectories.

## 1. Introduction

Alzheimer’s disease (AD), the leading cause of dementia, is characterized by the progressive accumulation of extracellular β-amyloid plaques and intracellular neurofibrillary tangles composed of hyperphosphorylated tau proteins (Scheltens et al., 2021). β-Amyloid plaques can be detected in the brain decades prior to symptom onset (Hansson, 2021; Palmqvist et al., 2017), and are detected in 20–40% of cognitively unimpaired elderly (Villemagne et al., 2013; Hedden et al., 2013; Jansen et al., 2015; Jack et al., 2017; Jansen et al., 2022). In contrast, tau neurofibrillary tangles bear a strong temporal and spatial link to neurodegeneration and cognitive decline (Villemagne et al., 2018; Bejanin et al., 2017). The formation of tau tangles, generally follows a hierarchical spatial progression, beginning in the transentorhinal cortex and spreading to the hippocampus, association cortices, and eventually to primary sensory regions (Braak and Braak, 1991). This stereotypical pattern of tau progression has been observed both postmortem (Scheff et al., 2006; Braak et al., 2011; Marquié et al., 2017; Ravikumar et al., 2024) and in vivo using tau positron emission tomography (PET) (Schöll et al., 2016; Johnson et al., 2016; Sanchez et al., 2021; Therriault et al., 2022; Leuzy et al., 2023; Ossenkoppele et al., 2025). However, more recent studies show that tau burden patterns exhibit notable heterogeneity across individuals with AD, often diverging from the stereotypical Braak staging system (Ferreira et al., 2020; Vogel et al., 2021; Young et al., 2022). Understanding the biological mechanisms underlying variation in individual spatial patterns of tau progression is crucial, as these may have significant clinical implications. For instance, both postmortem and clinical imaging studies have demonstrated that neocortical-predominant (hippocampal-sparing) tau-PET patterns are linked to earlier symptom onset and faster cognitive decline, while limbic-predominant patterns are associated with later onset and slower decline (Murray et al., 2011; Ossenkoppele et al., 2020). Inter-individual variations in premorbid brain structure are thought to be a key contributor to variations in tau spread (Jacobs et al., 2018; Bocancea et al., 2023), a notion that is especially supported by an accumulating body of studies that report strong associations between the spatial structure of brain networks and the trajectory of tau spread in AD (Hoenig et al., 2018; Ossenkoppele et al., 2019; Franzmeier et al., 2020; Vogel et al., 2020).

Connectome-based disease spreading models have emerged as a powerful framework for simulating tau propagation through human brain networks, incorporating both functional and structural connectivity in predicting tau distribution (Raj et al., 2021; Vogel et al., 2023). These group-level models have demonstrated that tau may spread along neuronal connections, measured by diffusion tensor imaging, and clarify how various brain regions contribute to tau propagation (Fornari et al., 2019). These findings support an influential hypothesis that tau propagates in a “prion-like” manner, spreading from an epicenter, typically in the (trans)entorhinal cortex, along synaptically connected regions, facilitated by the brain’s network structure (Clavaguera et al., 2009; de Calignon et al., 2012; Goedert et al., 2017; Kaufman et al., 2018). Human studies and animal models show greater tau accumulation in regions strongly connected to initially affected areas, indicating that tau spread follows functional and anatomical connectivity patterns (Liu et al., 2012; Frost and Diamond, 2010; Yu et al., 2021).

Resting-state functional MRI (fMRI) is a valuable technique for mapping brain functional architecture, particularly in deriving functional connectivity networks that capture coherent brain activity between regions. Several studies have shown that tau-PET patterns can be predicted or simulated using group-averaged functional connectivity networks (Sepulcre et al., 2017; Cope et al., 2018; Franzmeier et al., 2019, 2020, 2022; Ossenkoppele et al., 2019; Vogel et al., 2020, 2023; Ottoy et al., 2024; Wang et al., 2024), providing evidence for a potential link between functional connectivity and tau pathology. While averaging functional connectivity patterns across individuals creates a template connectome that captures stable patterns, it fails to account for individual connectivity differences. Functional connectomes retain unique, person-specific features that allow reliable fingerprinting of individuals (Finn et al., 2015; Amico and Goñi, 2018) throughout the AD continuum, and have therefore been suggested as a means to inform personalized models of disease progression (Stampacchia et al., 2024). Importantly, if the prion-like spread hypothesis holds (Cope et al., 2018; Bejanin et al., 2017; Tucholka et al., 2018), individual connectivity profiles should directly shape how tau spreads in each person. In other words, the specific architecture of a person’s brain network may determine not only the extent but also the pattern of tau propagation, influencing which regions become affected over time. Recognizing this individual-level variability is crucial, as it highlights connectivity not only as a population-level predictor but also as a potential determinant of personalized disease trajectories. Consistent with this, recent studies have begun to advocate for patient-tailored connectivity models to capture the heterogeneity of tau progression across individuals (Vogel et al., 2023; Tripathi et al., 2025), with evidence that clinical subtypes of AD, such as early- versus late-onset, show distinct patterns of both tau accumulation and functional connectivity (Schöll et al., 2017; La Joie et al., 2020; Lee et al., 2022; Franzmeier et al., 2018). At the same time, this does not preclude alternative explanations such as the role of selective regional vulnerability in modulating tau accumulation patterns (Leng et al., 2021; Kampmann, 2024; Xiao et al., 2025).

This study aims to determine whether individual-level FC, derived from resting-state fMRI, enhances the characterisation of individual tau-PET patterns beyond what is achievable using group-level template FC. We hypothesize that individual FC patterns can account for more of the variance observed in tau-PET patterns across subjects compared to group-averaged connectivity. Importantly, we investigate this across multiple spatial resolutions (from 100 to 1000 cortical regions) using subject-specific surface-based parcellations, enabling multiscale modelling of both FC and tau-PET. We further investigate whether FC more accurately reflects individual tau-PET distributions than canonical group-based on- and off-target binding patterns. Additionally, we test the specificity of this relationship by assessing whether the unique explanatory power of individual FC for tau is absent in β-amyloid, for which the prion-like spread hypothesis is not assumed. Finally, we test whether baseline FC is associated with future tau accumulation, thereby examining its potential as a prognostic biomarker. Overall, our approach moves beyond the traditional reliance on group averages and the use of a single spatial scale, shedding light on how individual variations in functional connectivity across scales contribute to the spatial distribution of tau pathology in patients along the AD continuum.

## 2. Results

Our study included 805 elderly participants (mean [std]: 71 [9] years; 55% females) from the Swedish BioFINDER-2 study (https://biofinder.se/). Among them, 398 were cognitively normal (CN), 77 reported subjective cognitive decline (SCD), 156 were diagnosed with mild cognitive impairment (MCI), and 174 were diagnosed with AD dementia (ADD). All patients with ADD, MCI, and SCD, and a subset of the CN (n=89, 22.4%) were Aβ-positive based on CSF testing. Demographic information for each group is shown in Table 1. The data for each participant included structural and resting-state functional MRI and tau-PET. Aβ-PET was available for CN, SCD, and MCI participants but not for ADD patients. Furthermore, our longitudinal analysis included follow-up tau-PET scans from a subset of the participants (n=422, 52.4% of sample) with a mean follow-up time of 1.8±0.48 years.

### 2.A. Patient-specific FC improves the explanation of individual tau-PET patterns beyond template FC

**Fig. 1 |:**
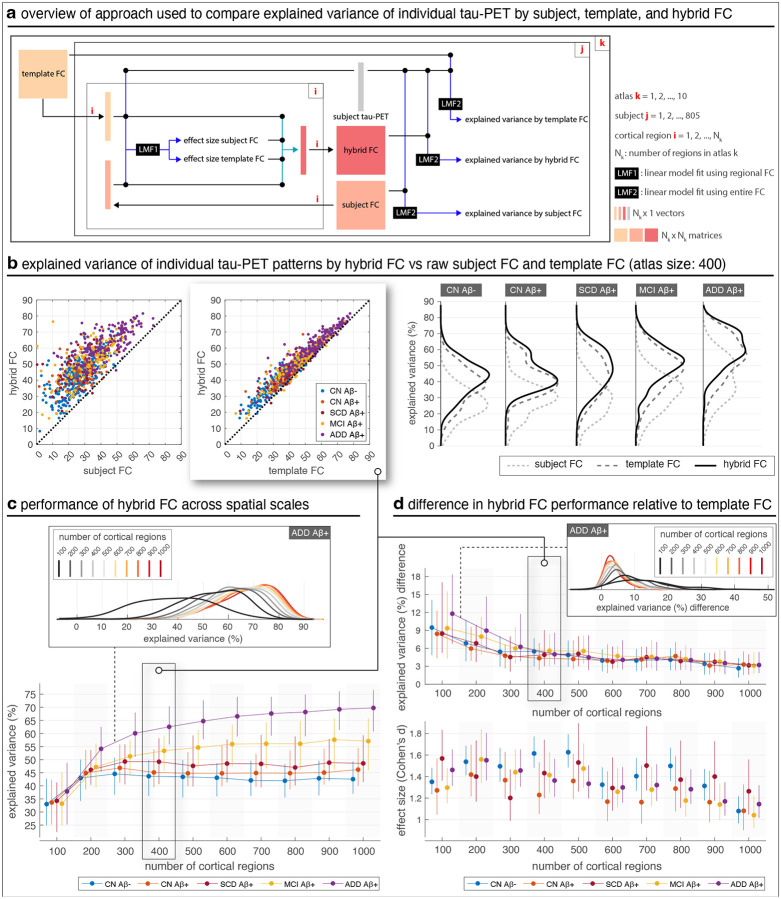
Hybrid FC explains individual tau-PET better than raw subject FC and template FC, across spatial scales. (a) Overview of the approach used to evaluate additional power of hybrid FC beyond template FC in explaining individual tau-PET. (b) Axis values are the coefficient of determination (R^2^, in percentage) of individual tau PET. Axis labels specify regressor(s) used. Each marker represents a subject. Marginal distributions for the groups are shown as kernel density plots across the different FC types. FC matrices were constructed using cortical regions defined by Schaefer atlas (Schaefer et al., 2018) using 400 cortical regions. All reported R^2^ are corrected R^2^ to ensure unbiased comparison of models with different numbers of regressors. (c) At later stages of disease (MCI and ADD), explained variance increases when using FC built with more fine-grained cortical regions. For the AD Dementia group, marginal distributions of the data are compared. (d) Across all disease stages, additional explained variance of hybrid FC over template FC reduces as a function of spatial granularity (top) but the effect size remains consistently strong (bottom); for the AD Dementia group, marginal distributions of the data in the top panel are shown. Whiskers in the top plot represent the 25th to 75th percentiles; markers indicate the median. Whiskers in the bottom plot show the 95% confidence interval of Cohen’s *d*; the difference between the two models for each disease stage and at each spatial was significant based on paired t-test, all p-values < 1e-4. CN: cognitively normal; SCD: subjective cognitive decline; MCI: mild cognitive impairment; ADD: Alzheimer’s disease dementia.

Using resting-state fMRI, we derived both group-level and subject-specific connectivity profiles, providing the basis for subsequent analyses. To obtain a holistic estimate of the added explanatory power of subject-specific FC beyond template FC, we constructed an individualized “hybrid FC” for each subject that integrated both shared and subject-specific connectivity features (Fig. 1a). Across the disease continuum, hybrid FC consistently outperformed both template and raw subject FC in explaining tau-PET patterns (Fig. 1b), with particularly strong effects in symptomatic groups where tau progression is most advanced. The total explained variance by the hybrid FC model largely depended on the spatial granularity of the design: for example, in patients with ADD, the median ranged from 40% to 70% (Fig. 1c). While template FC also improved with finer parcellations (Fig. S1b), hybrid FC consistently achieved higher explanatory power across all groups and scales. In contrast, raw subject FC showed weaker performance, except in the ADD group, highlighting the stabilizing role of combining subject- and group-level information. Across groups, while the absolute difference in explained variance between hybrid and template FC gradually declined with increasing resolution (Fig. 1d, top), the difference relative to raw subject FC remained largely stable (Fig. S1c), even increasing slightly at higher resolutions in later-stage groups. The effect size of hybrid FC’s advantage over template FC also declined somewhat with scale but remained very strong (Cohen’s *d* > 1) across a broad range of atlas sizes, only falling slightly below this threshold at the highest resolutions (Fig. 1d, bottom; Fig. S1e–f). These results demonstrate that hybrid FC provides a robust and scalable framework for capturing both canonical and individualized contributions to tau-PET patterns, particularly in later disease stages.

**Fig. 2 |:**
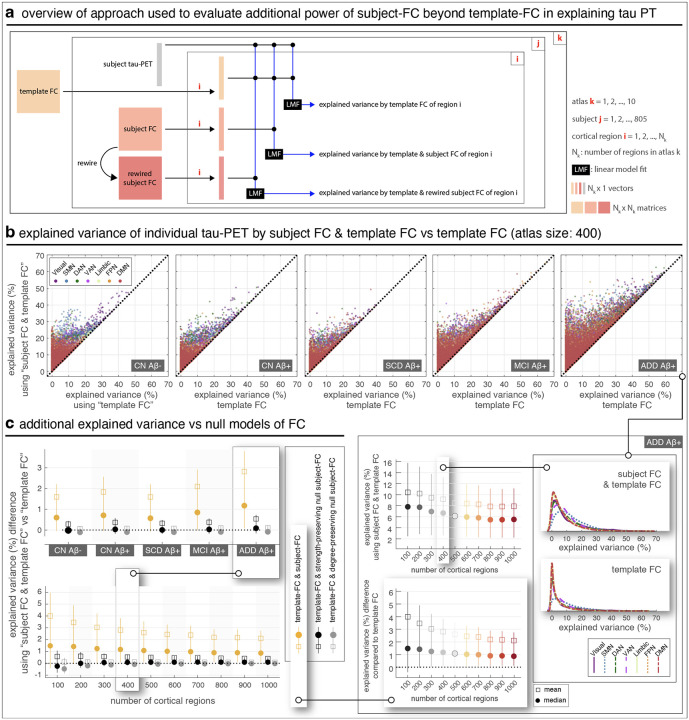
Regional subject FC provides additional explanatory power in explaining individual tau-PET beyond template FC, across spatial scales. (a) Overview of the approach used to evaluate the additional power of subject FC beyond template FC in explaining regional tau-PET patterns. By “explained variance”, we refer to the coefficient of determination (R^2^) from model fits; throughout this work, we report corrected R^2^ values to ensure unbiased comparison between models with differing numbers of regressors. In this figure, the model using only template FC includes one regressor, whereas the model using both template FC and subject FC includes two. Rewired null models were used to assess the importance of higher-order topological structure in subject FC. (b) Comparison of explained variance when using both subject FC and template FC versus using only template FC. The scatter plots show explained variance for individual regions and subjects. More detailed comparisons are shown in the inset on the bottom-right for group ADD and in Fig. S2a–b for the other groups. Whiskers represent the 25th to 75th percentiles, and markers indicate the mean and median, computed across regions and subjects. (c) Additional explained variance from including subject FC, compared to two null models where subject FC was rewired to preserve either nodal degree (Maslov and Sneppen, 2002) or both nodal degree and strength (Milisav et al., 2025). Whiskers represent the 25th to 75th percentiles, and markers indicate the mean and median, computed across regions and subjects. CN: cognitively normal; SCD: subjective cognitive decline; MCI: mild cognitive impairment; ADD: Alzheimer’s disease dementia; SMN: somatomotor network; DAN: dorsal attention network; VAN: ventral attention network; FPN: frontoparietal network; DMN: default mode network.

These whole-brain results were motivated by regional analyses in which we first asked whether subject FC provides additional explanatory power beyond template FC at the level of individual regions (Fig. 2a). Using cortical atlases with varying parcellation sizes (100 to 1000 regions), we regressed each subject’s tau-PET signal on both template and subject FC, and compared explained variance across disease groups. Across scales, the inclusion of subject FC consistently improved model fits relative to template FC alone, with the additional variance explained being most pronounced in later disease stages (Fig. 2b). The explained variance per region decreased with increasing atlas size, since regions became smaller, however, this decline was less steep than the decrease in region size itself, indicating a net gain in cumulative variance explained across regions (Fig. 2b, bottom; Fig. S2b). To determine whether these effects were attributable to higher-order network topology rather than trivial graph properties, we compared against null models where subject FC was rewired while preserving either nodal degree or both degree and strength (Maslov and Sneppen, 2002; Milisav et al., 2025). The additional explained variance from subject FC exceeded both null models (Fig. 2c; Fig. S2c), confirming that the contribution of subject-specific FC is meaningful and not simply a byproduct of low-level graph features. Importantly, these regional analyses provided the regression weights that formed the basis for hybrid FC, linking the mechanistic justification for its construction with the robust gains observed in whole-brain models.

To assess whether these effects differed across regions associated with different canonical functional networks, we conducted complementary analyses at both the regional and network levels (Fig. S3 and Fig. S4, respectively). Across disease stages, the Default Mode (DMN) and Frontoparietal (FPN) networks consistently showed the strongest associations with tau-PET patterns, while the limbic network was more prominent in earlier, Aβ+ groups. Performance in networks such as the visual and limbic tended to plateau or decline at finer parcellation scales, whereas the DMN and FPN remained robust. These convergent findings across regional and network analyses reinforce the central role of DMN and FPN in capturing tau-related variability, while highlighting that other networks may be more sensitive to spatial granularity.

### 2.B. Hybrid FC better explains individual tau-PET than cohort-level canonical tau-PET patterns or individual Aβ-PET patterns, alone or combined

The results thus far have demonstrated that individual FC provides additional explanatory power beyond group-level template FC in predicting individual tau-PET patterns. We next sought to determine whether, and to what extent, FC captures unique aspects of individual tau-PET patterns beyond what can be explained by canonical tau-PET patterns, addressing a dimension that has received limited attention in previous studies. Here, canonical patterns refer to stereotypical spatial distributions of tau-PET signal across the cortex, which we derived by estimating ensemble off- and on-target tau-PET retention patterns present across the entire cohort via Gaussian mixture modelling (S5–S7). These patterns represent generalizable features of tau accumulation, distilled across individuals, and serve as a reference or baseline model of tau-PET topography.

**Fig. 3 |:**
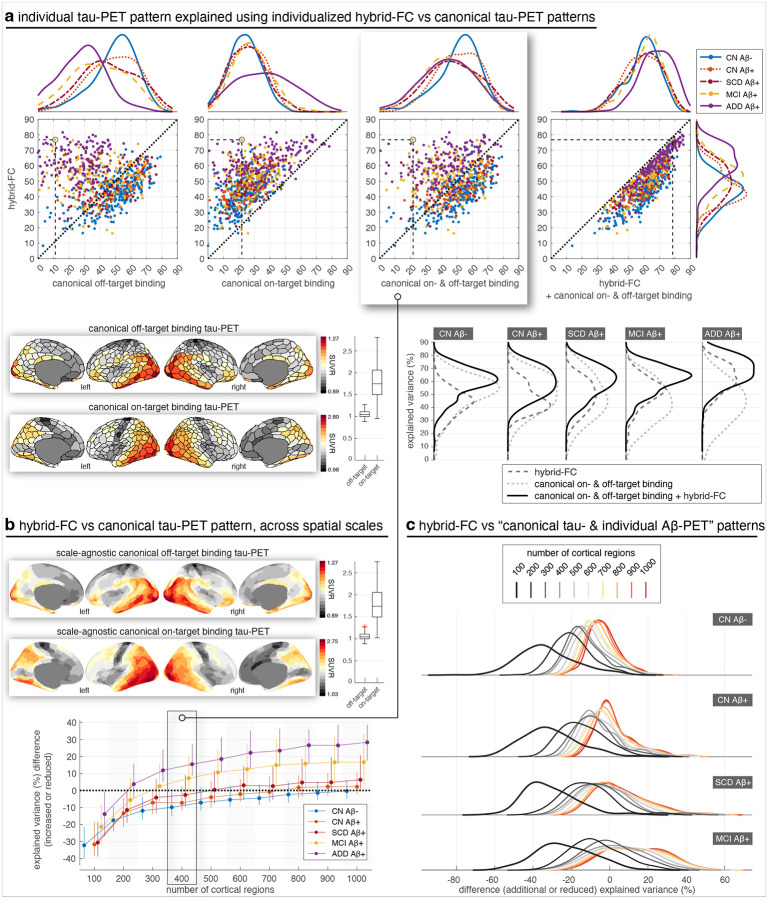
Hybrid FC explains individual tau-PET better than canonical tau-PET patterns, across spatial scales. (a) Axis values are explained variance (corrected R^2^, in percentage) of individual tau PET. Axis labels specify regressor(s) used. Each marker represents a subject; e.g. for the circled Aβ+ patient with MCI, hybrid FC (y-axis across all 4 panels) explains 77% of the spatial variance in their tau-PET pattern whereas canonical off- (1st panel, x-axis) and on-target (2nd panel, x-axis) binding explain 11% and 22% of the variance, respectively; canonical off- and on-target binding together (3rd panel, x-axis) explain 22% of the variance whereas if hybrid FC is also used (4th panel, x-axis) the explained variance increases to 79%. Marginal distributions for the groups are shown as kernel density plots; in the bottom plot, marginal distributions shown next to scatter plots (top panel) are re-configured (bottom panel) to enable comparison of kernel densities associated to each group for the different regressors. FC matrices were constructed using cortical regions defined by Schaefer atlas (Schaefer et al., 2018) using 400 cortical regions. All reported R^2^ are corrected R^2^ to ensure unbiased comparison of models with different numbers of regressors, across this study. (b) The performance of hybrid FC over canonical tau-PET patterns further improves as a function of the resolution of FC, where FC build using more fine-grained functional regions performs better. The cortical projections show the scale-agnostic version of the off- and on-target tau PET binding patterns obtained by averaging the binding patterns derived for the ten different partial scales; the off- and on-target tau binding patterns for each spatial resolution are shown in Fig. S6 and Fig. S7 in the supplementary material, respectively. Whiskers represent the 25th to 75th percentiles; markers indicate the median. The marked panel (i.e. for 400 regions) relates to data shown in the third, top panel in (a). (c) The difference in explained variance of individual tau-PET by hybrid FC relative to canonical tau-PET patterns combined with individual Aβ-PET is shown across spatial scales. The difference in performance increases as the spatial resolution increases across the three groups, with hybrid FC showing improved performance (values above zero) most dominantly in Aβ+ patients with MCI and at high spatial resolutions (parcellations with 400 cortical regions and above). The differences are generally greater in Aβ+ patients with MCI. CN: cognitively normal; SCD: subjective cognitive decline; MCI: mild cognitive impairment; ADD: Alzheimer’s disease dementia.

When comparing model performance, we found that for Aβ+ individuals with MCI or ADD, hybrid FC explained individual tau-PET patterns better than canonical tau-PET patterns, while in early-stage patients, whose tau pathology is more spatially restricted, canonical tau-PET patterns provided a better explanation (Fig. 3a). However, this distinction shifted when we increased the spatial resolution of the analysis, i.e., using more fine-grained cortical regions (Fig. 3b). First, at higher resolutions of cortical parcellation (>300 regions), hybrid FC more distinctly outperformed canonical patterns in Aβ+ patients with MCI or ADD, and second, it matched or outperformed canonical patterns even in early disease stages. These findings suggest that individual FC may capture subtle, spatially localized features of tau distribution that are not reflected in cohort-level canonical patterns, particularly when analysed at higher spatial granularity. Furthermore, across all individuals, combining canonical patterns with hybrid FC explained more variance in individual tau-PET patterns than either factor alone, suggesting that the two provide complementary information about the spatial distribution of tau pathology (Fig. 3a, top-right). These trends were further supported by results across spatial scales (Fig. S8). While canonical tau-PET patterns (especially off-target binding) consistently declined in predictive power with increasing atlas resolution (Fig. S8a–c), hybrid FC performance increased (Fig. 2c), eventually surpassing the canonical models at finer scales. Direct comparisons confirmed that the added value of hybrid FC over canonical patterns grew with spatial granularity, particularly in Aβ+ individuals with MCI and ADD (Fig. S8e–f). These findings reinforce that canonical tau-PET patterns and individual FC capture different aspects of individual tau-PET, and that the benefit of FC-based modelling becomes increasingly evident when leveraging fine-grained spatial information.

For CN individuals and patients with MCI who had Aβ-PET scans available, we further explored whether using individual Aβ-PET patterns as regressors in addition to canonical tau-PET patterns improves performance relative to that provided by hybrid FC. The results revealed that individual Aβ-PET patterns explained individual tau-PET patterns only minimally, compared to hybrid FC and canonical tau-PET patterns (Fig. S9). Even when combined with canonical tau-PET patterns they only marginally increased the performance in Aβ+ patients with MCI while the performance for the majority of Aβ+ patients with MCI remained below that of hybrid FC (Fig. 3c). Together, these results underscore the unique and complementary value of individualized FC in capturing personalized features of tau pathology that are not fully accounted for by canonical tau or amyloid-based models.

### 2.C. Hybrid FC better captures individual tau-PET but not Aβ-PET compared to canonical PET patterns

**Fig. 4 |:**
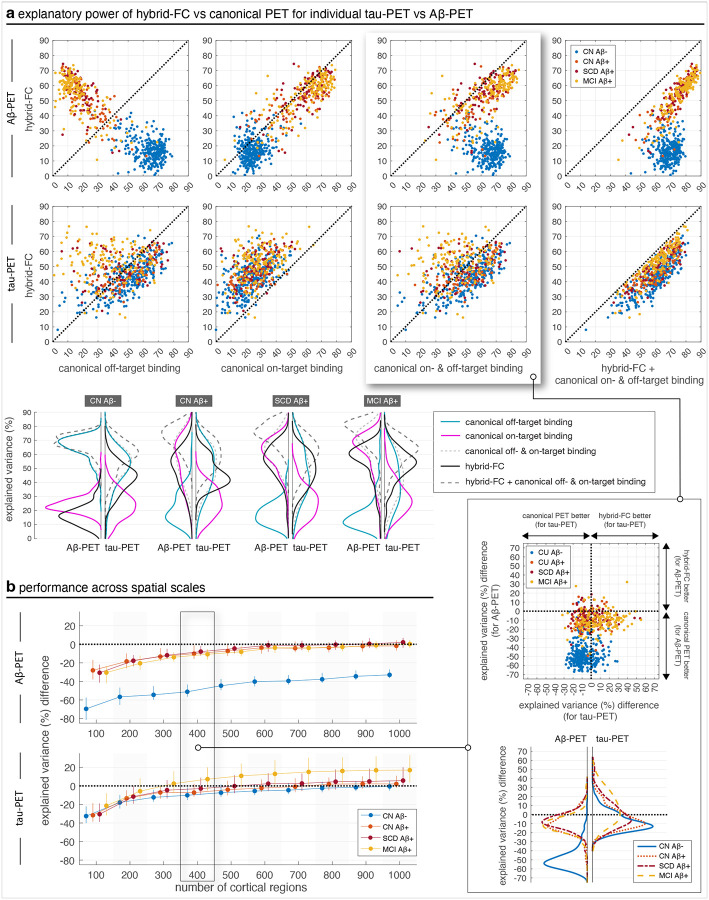
Hybrid FC explains individual tau-PET, but not Aβ-PET, better than canonical PET patterns. (a) In the first row, linear models are fit to individual Aβ-PET patterns using different regressors as specified by axes labels of each scatter plot. Each marker represents a subject and axes values are the percentage of explained variance (corrected R^2^ resulting from linear fit models). In the second row, linear fit models are instead fitted to individual tau-PET patterns. Marginal distributions of explained variance are compared across groups. Note that in each group explained variances are being compared for tau-PET and Aβ-PET which are different in nature in terms of the amount of spatial content they entail (Aβ-PET patterns generally exhibit less spatial complexity compared to tau-PET patterns), and, as such, comparison between the two should be made in relation to difference in performance using different regressors for each data modality (i.e., up-down shift in kernel densities for each modality) rather than comparing explained variance of tau-PET to that of Aβ-PET. (b) Explained variance using hybrid FC minus explained variance using canonical PET patterns across scales. Differences in explained variance for the data in the first, second, and fourth columns in (a), across spatial scales, are shown in Fig. S10, whereas the absolute explained variances are shown in Fig. S11. Whiskers represent the 25th to 75th percentiles; markers indicate the median. The marked panel (i.e. for 400 regions) relates to data shown in (a). CN: cognitively normal; SCD: subjective cognitive decline; MCI: mild cognitive impairment.

The key focus of this study has been to understand the explanatory power of FC in relation to individual tau-PET patterns, an endeavour motivated by the hypothesis that tau pathology spreads along neuronal communication pathways. To test the specificity of this hypothesis for tau pathology, we examined whether, and if so to what degree, FC contributes to explaining individual Aβ-PET patterns, for which this hypothesis is less commonly proposed. We therefore repeated the analysis with Aβ-PET data and compared the performance to the tau-PET analysis within the same individuals; patients with ADD excluded as they lacked Aβ-PET data. The superior explanatory power of hybrid FC over canonical PET patterns we observed for tau-PET was not observed for Aβ-PET (Fig. 4a). Compared to the explanatory power of hybrid FC, Aβ-PET patterns were better explained by the estimated canonical off-target binding Aβ-PET pattern in Aβ- individuals, and by the canonical on-target binding pattern in Aβ+ patients. This contrasts with observations for individual tau-PET patterns. To further contextualize these findings, we compared marginal distributions of explained variance. This revealed that hybrid FC largely outperforms canonical patterns for tau-PET, but not for Aβ-PET (Fig. 4a, bottom-right inset). The difference increased as the spatial granularity of the analysis increased (Fig. 4b). For Aβ-CN and Aβ+ patients with MCI, the difference in explained variance was consistently greater for tau-PET compared to Aβ-PET, whereas for Aβ+ CN the performances were on par. Furthermore, comparison of differences in explained variances for other settings (e.g. hybrid FC minus on-target binding pattern) show that canonical patterns explain more variance in Aβ-PET than hybrid FC, especially in Aβ+ CN and patients with SCD or MCI (Fig. S10b–c), while the reverse is true for tau-PET. This trend is reinforced by the absolute explained variances (Fig. S11), where canonical Aβ-PET patterns consistently explain more variance than hybrid FC, in contrast to tau-PET where hybrid FC explains the most variance across all groups and scales. This analysis confirmed the specificity of the communication pathway hypothesis for tau, as FC-based models outperformed canonical patterns for tau-PET but not for Aβ-PET, where the latter were better explained by cohort-derived spatial PET patterns included in our modelling framework.

### 2.D. Longitudinal tau-PET is better explained by Hybrid FC than template FC

**Fig. 5 |:**
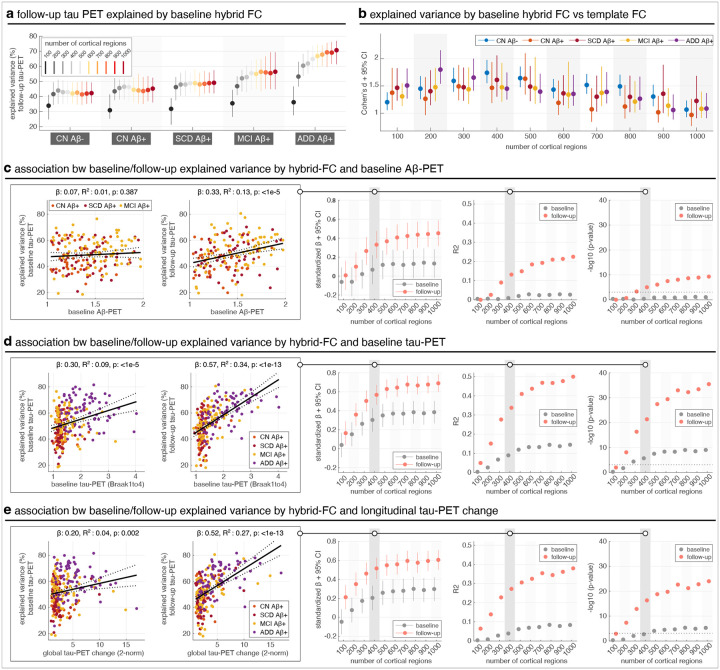
Explained variance of follow-up individual tau-PET patterns using baseline hybrid FC. (a) Baseline hybrid FC explained individual follow-up tau-PET patterns across clinical groups, with performance increasing at finer spatial scales and plateauing around 400–600 cortical regions. (b) Across all scales and groups, hybrid FC outperformed template FC in explaining follow-up tau-PET, as quantified by Cohen’s *d*. (c–e) Associations between tau-PET model performance (explained variance, EV) and baseline biomarkers: global Aβ-PET SUVR (c), baseline tau-PET (Braak I–IV) (d), and longitudinal tau-PET change (Braak I–IV) (e); for Aβ-PET, the meta ROI SUVR is the average SUVR calculated from a global neocortical ROI, including prefrontal, lateral temporal, parietal, anterior cingulate and posterior cingulate/precuneus. For each biomarker, we modelled its relationship with tau-PET EV at both baseline and follow-up using linear regression with age and sex as covariates. Predictors and outcomes were z-scored, and standardized beta coefficients (with 95% CI), R^2^, and p-values were extracted across spatial scales. Leftmost plots in each panel show associations for one representative spatial scale (400 regions); rightmost columns summarize results across scales. Analyses were restricted to Aβ-positive individuals (CN Aβ+, SCD Aβ+, MCI Aβ+, ADD Aβ+), except for Aβ-PET analyses (panel c), where the ADD group was excluded due to missing Aβ-PET data. The CN Aβ- group was excluded from all analyses in panels c-e to avoid skewing the regression due to consistently low biomarker levels in this group.

For a subset of patients with available follow-up tau-PET scans (52% of patients, see Table 1), we examined the extent to which hybrid FC derived from baseline data could predict the individual’s future tau-PET patterns. Hybrid FC explained follow-up tau-PET with increasing accuracy at finer spatial scales, plateauing around 400–600 regions (Fig. 5a), and consistently outperformed template FC across all clinical groups and spatial resolutions (Fig. 5b). While Fig. 5a–b focus on follow-up tau-PET, we confirmed that hybrid FC achieved similar levels of explained variance for baseline tau-PET patterns in groups consisting of the same matching individuals (Fig. S12a), reinforcing the model’s robustness across timepoints. Moreover, hybrid FC provided additional predictive value over template FC, particularly at finer spatial resolutions and in later disease stages (Fig. S12b). However, despite this group-level consistency, individual-level comparisons revealed substantial variability: the correlation between how well hybrid FC explained baseline versus follow-up tau-PET patterns was modest (Fig. S12c, left), with standardized effect sizes peaking at intermediate spatial resolutions (Fig. S12c, middle). This suggests that, while hybrid FC captures stable spatial features at the cohort level, individual tau-FC alignment is dynamic over time, likely reflecting subject-specific progression of tau along functional pathways.

To better understand the factors shaping this heterogeneity, we next examined whether baseline aggregate pathological markers were associated with the model’s ability to explain tau-PET patterns at either timepoint. Explained variance at follow-up was only weakly associated with baseline Aβ-PET levels (Fig. 5c, left), and this relationship was not significant at baseline (Fig. 5c, middle/right). In contrast, baseline tau-PET levels were more strongly associated with the explained variance of follow-up tau-PET, and this relationship was stronger than at baseline across most spatial scales (Fig. 5d). These patterns suggest that hybrid FC may capture aspects of functional architecture that interact with the spatial distribution of existing tau burden to shape where tau pathology subsequently accumulates. To clarify why the association between baseline aggregate tau burden and explained variance was stronger at follow-up, we tested baseline Aβ-PET as a mediator across spatial scales (Fig. S13). Mediation was evident only for follow-up, becoming significant at higher spatial scales, and was absent for baseline at all scales. In line with this interpretation, subjects who showed greater longitudinal changes in tau-PET (i.e., greater tau spread) also exhibited higher follow-up explained variance (Fig. 5e), again more so than at baseline, and with the effect increasing and stabilizing across spatial granularity. Together, these findings reinforce the potential predictive utility of hybrid FC while also offering indirect support for models of tau propagation involving transneuronal spread along functionally connected pathways. By linking the structure of baseline FC to future patterns of tau accumulation, our results highlight a potential mechanism through which individual FC may constrain or guide the spread of tau pathology across the cortex.

## 3. Discussion

Network-based models of tau propagation propose that the spread of pathology in Alzheimer’s disease (AD) follows the brain’s intrinsic connectivity architecture, yet direct evidence at the individual level has been limited. We hypothesized that subject-specific functional connectivity (FC) would shape the spatial distribution of tau pathology, such that individual differences in network organization would account for corresponding differences in tau-PET patterns. Using 1227 tau-PET scans from 805 participants in the BioFINDER-2 study, we found that individual-level FC indeed improved the explanation of tau-PET topographies compared to group-level FC alone, indicating that both shared and idiosyncratic features of network architecture jointly contribute to tau deposition. These effects were particularly evident in symptomatic individuals, where tau progression is most advanced, and at higher spatial resolution. Furthermore, baseline FC predicted future tau deposition, reinforcing its role as a marker of disease progression. Taken together, these findings provide some of the strongest evidence to date for the network spread hypothesis: idiosyncrasies in individual network architecture translate into variability in tau distribution, above and beyond what can be explained by canonical connectivity or PET patterns. This work thus reframes our understanding of tau propagation in AD, underscoring that individual variability in functional brain architecture is not noise, but a key determinant of disease expression across individuals.

Our results advance and refine the prion-like propagation hypothesis of tau pathology, which posits that tau spreads transneuronally along functional and structural pathways. While previous studies have shown that group-averaged connectomes strongly predict stereotypical patterns of tau spread (Franzmeier et al., 2020; Vogel et al., 2020), these models do not accurately capture subject-specific heterogeneity of tau deposition. Here, we show that individualized FC accounts for variance in regional tau burden that is not explained by template FC (Fig. 2), especially in later disease stages. The explanatory gain of subject-specific FC over degree- and strength-preserving null models suggests that it is not merely basic topological properties but higher-order network organization that shapes tau spread (Fig. 2c). These findings underscore the importance of considering personalized connectomic architecture to understand individual differences in tau distribution, building on fMRI-based evidence that patients with AD show individual-specific functional connectivity profiles (Millar et al., 2020; Stampacchia et al., 2024), with variability across networks such as the default mode, salience, and frontoparietal control (Adriaanse et al., 2014; Gour et al., 2014; Lehmann et al., 2015; Lin et al., 2018; Franzmeier et al., 2018; Tu et al., 2024). Together, our results highlight that variability in brain connectivity is not noise, but a contributor to the unique manifestation of tau pathology in each patient.

Notably, subject-specific FC predicted tau burden but not Aβ-PET patterns (Fig. 4a–b), suggesting relative specificity to tau, and reinforcing the notion that FC is more tightly linked to the intracellular dynamics of tau rather than extracellular Aβ. Furthermore, FC explained individual tau-PET heterogeneity in later disease stages better than cohort-derived canonical tau-PET patterns (Fig. 3b), even when the latter were combined with individual Aβ-PET data (Fig. 3c). These findings raise important mechanistic questions about upstream modulators of FC, such as Aβ pathology, which accumulates early in the disease and may shape the functional connectome in ways that influence future tau spread. Consistent with this, we found that baseline Aβ-PET levels were more strongly associated with the ability of baseline FC to explain future tau-PET patterns than concurrent tau-PET patterns, even though both tau scans were predicted using the same FC data (Fig. 5c). This suggests that Aβ-related changes to functional connectivity may act as a priming mechanism, rendering certain pathways more permissive to subsequent tau propagation. Mediation analyses revealed that baseline Aβ explains part of the association between early tau burden and future FC-constrained tau patterns, but not concurrent patterns, with the mediated component becoming more apparent at finer parcellations (Fig. S13). This scale dependence suggests that Aβ-related influences on tau spread are expressed at meso-scale functional architecture captured by higher-resolution FC, while a substantial direct effect of baseline tau remains. Notably, this effect was not driven by evolving FC profiles, but rather by baseline Aβ influencing the extent to which baseline FC predicts longitudinal changes in tau burden. These findings corroborate prior work showing that Aβ deposition can induce functional hyperconnectivity or dysregulation that facilitates tau spread (Jones et al., 2017; Palmqvist et al., 2017; Franzmeier et al., 2020; Pereira et al., 2019; Roemer-Cassiano et al., 2025), and they extend this notion by suggesting a temporally layered mechanism in which early Aβ accumulation influences later FC-constrained tau propagation. While our analyses focused on static FC, future efforts integrating subject-specific Aβ maps and dynamic FC may help determine whether Aβ actively modifies the functional connectome to accelerate tau transmission. Together, these findings support a pathology-driven, individualized framework for tau propagation.

Our findings highlight spatial scale as a critical factor in modelling the relationship between functional connectivity (FC) and tau pathology. While prior studies have used parcellations of 400 regions or fewer, we systematically evaluated FC and tau-PET associations across ten parcellation resolutions ranging from 100 to 1000 regions, enabling multiscale analysis of both modalities. Across analyses, higher-resolution parcellations consistently improved model performance, particularly in symptomatic individuals, where tau is more diffusely distributed and more variable (Fig. 2c, top; Fig. 5b). For example, in patients with ADD, the median explained variance using hybrid FC increased from approximately 38% at 100 regions to nearly 70% at 1000 regions. This scale-dependent improvement was also evident when comparing hybrid FC to canonical tau-PET patterns (Fig. 3b) and Aβ-PET patterns (Fig. 4b), and persisted even after controlling for model complexity via null models (Fig. 2c). Although the absolute relative advantage of hybrid over template FC decreased with increasing resolution due to performance saturation in subject FC models, particularly in CN and MCI (Fig. S1a), the difference remained positive and statistically strong across scales (Fig. 2d). These observations reflect a broader methodological point: coarser atlases introduce greater spatial averaging, obscuring local variation in both FC and tau-PET maps. To address this, we implemented subject-specific surface-based parcellation using the Schaefer atlas, applied in each subject’s native anatomical space. All PET and fMRI data were co-registered to the individual’s T1-weighted image and projected onto native cortical surfaces, avoiding warping to template spaces (e.g., MNI) and preserving fine-grained anatomical detail. This approach enabled accurate parcellation at high spatial resolutions and ensured consistency between modalities, resulting in richer input data for modelling. Importantly, if tau pathology indeed spreads in a prion-like manner along brain networks, then modelling this process at coarse spatial scales—where each region encompasses broad, heterogeneous cortical territories—is unlikely to reveal the specific propagation dynamics underlying individual variability. Notably, the use of hybrid FC, which combines subject-specific and template-level FC, helps mitigate the increased spatial noise and instability that can arise when estimating fine-grained FC matrices at the individual level, offering a stable yet personalized model that retains subject-specific features while enhancing signal reliability. Our results suggest that biologically meaningful differences in connectivity–tau coupling emerge more clearly at finer scales, supporting the idea that spatial granularity, and individualized modelling, are essential parameters for advancing connectome-based models of tau spread.

Building on the strong relationship between individual functional connectivity (FC) and tau pathology, this work lays the groundwork for the development of personalized, network-aware models of tau propagation that may advance prognostic precision and individualized intervention strategies in Alzheimer’s disease. By showing that subject-specific FC robustly explains individual tau-PET patterns, and outperforms group-average and canonical models, particularly at finer spatial scales, we lay the groundwork for future applications in early-stage risk profiling and personalized disease monitoring. Notably, the Default Mode and Frontoparietal networks consistently exhibited the highest explained variance across disease stages, emphasizing their centrality as vulnerable circuits where individual FC profiles most sensitively relate to tau accumulation. Importantly, these are also networks characterized by high inter-individual variability (Mueller et al., 2013; Finn et al., 2015), suggesting that their role as epicentres of tau spread may be precisely where subject-specific FC provides the greatest added value. These results suggest that individualized FC patterns capture not only global propagation routes, but also regional susceptibility linked to network architecture. Building on this, a critical next step will be integrating subject-specific FC with biological markers of regional vulnerability—such as excitatory neuron density (Fu et al., 2019; Leng et al., 2021), myelination gradients (Rubinski et al., 2022; Depp et al., 2023), microglial activity (Anand et al., 2022), and regionally patterned gene expression (Montal et al., 2022; Yu et al., 2024; Anand et al., 2024)—to better model why certain brain regions serve as preferential sites for tau accumulation (Xiao et al., 2025). This would allow hybrid models to not only simulate how tau spreads across an individual’s network but also estimate where it is most likely to emerge or intensify. While the integration of such multimodal biological features lies beyond the scope of the present study, our individualized FC framework provides a scalable and biologically grounded platform for this endeavour. Together, these findings support a precision connectomics approach to AD, with the potential to transform prediction and monitoring of tau spread and, ultimately, guide individualized therapeutic strategies to intercept its progression.

Key strengths of our study include its sample size, longitudinal design, and multimodal characterization using PET and fMRI, as well as the use of precision-connectomics methods that account for individual variability at multiple spatial scales. However, several limitations should be acknowledged. First, FC estimated via resting-state fMRI is an indirect proxy for neuronal signalling and is sensitive to physiological noise, dynamic fluctuations, and scanner artifacts (Logothetis, 2008; Leonardi and Van De Ville, 2015; Lurie et al., 2020). It also fails to distinguish direct from polysynaptic pathways (Honey et al., 2009). These issues may reduce the mechanistic interpretability of FC as a marker of directed communication, especially given cautions against overinterpreting static FC estimates in isolation, as dynamic FC measures may conflate true neural variability with confounding sources (Liegeois et al., 2017; Lurie et al., 2020). Nonetheless, the correspondence between our findings and results from animal models (Basheer et al., 2024), in which microscale neuronal dynamics can be more directly assessed, suggests a degree of cross-species consistency in the observed role of connectivity in tau spread. Second, while we demonstrate that subject-specific FC explains tau burden, we did not explicitly distinguish between tau presence and load (Vogel et al., 2020; Xiao et al., 2025) in our models, nor did we incorporate orthogonal biological features that could modulate vulnerability, such as gene expression or blood flow. Third, limited spatial resolution of PET imaging (compared to MRI) may introduce noise, especially at higher granularity, though our results suggest that a biologically meaningful spatial pattern remains detectable. Fourth, although FC captures aspects of communication relevant to tau spread, structural connectivity (SC) offers a more direct account of axonal pathways and may be especially informative in early disease stages (Liu et al., 2012; de Calignon et al., 2012; Vogel et al., 2020; Raj et al., 2021; Vogel et al., 2023; Xiao et al., 2025). Though subject-specific SC modelling remains technically difficult to robustly estimate from diffusion MRI at the clinical scale, our individualized FC framework lays the groundwork for future multimodal models that integrate both functional dynamics and structural constraints, potentially yielding more mechanistic insight into the spread of tau pathology. Finally, while our focus was on cross-sectional FC, longitudinal changes in FC, and their relationship to tau spread, remain an important future direction to establish temporal dynamics and causal relationships.

In conclusion, our findings establish that subject-specific functional brain architecture plays a crucial role in shaping the spatial distribution and progression of tau pathology in Alzheimer’s disease. By demonstrating that individual FC improves explanatory power beyond template and canonical tau models, and predicts future tau deposition, our work provides strong evidence for the network spread hypothesis and lays the foundation for a precision biomarker framework. Future research should integrate individual Aβ patterns, structural connectivity, and regionally specific vulnerability factors to refine mechanistic models of tau spread. Moreover, extending these models to capture the distinction between tau presence and load, and testing directional, activity-based mechanisms, will be essential not only for elucidating disease biology but also for advancing individualized biomarker staging and targeted interventions aimed at intercepting tau propagation before widespread cortical involvement occurs.

## 4. Methods

### Participants

We used data from the Swedish BioFINDER-2 study (NCT03174938). Information regarding recruitment, diagnostic criteria, and Aβ positivity assessment for the BioFINDER-2 cohort (https://biofinder.se/) has been described in detail previously (Leuzy et al., 2020; Palmqvist et al., 2020). All participants provided written informed consent in accordance with the Declaration of Helsinki. Ethical approval for the study was obtained from the Ethics Committee of Lund University, Lund, Sweden, with additional permissions for PET imaging granted by the Swedish Medicines and Products Agency and the local Radiation Safety Committee at Skåne University Hospital, Sweden.

Our study cohort included 805 participants aged 50+, consisting of 398 cognitively normal (CN), 77 with subjective cognitive decline (SCD), 156 with mild cognitive impairment (MCI), and 174 with AD dementia (ADD). Individuals with SCD, MCI, or ADD were all Aβ+ based on CSF testing whereas the CN individuals were split into two groups based on their CSF Aβ status; a pre-established cutoff of 0.08 on the CSF Aβ42/Aβ40 ratio was used to define Aβ positivity based on Gaussian mixture modeling (Palmqvist et al., 2020), and CSF Aβ42 and Aβ40 were measured using the Elecsys immunoassays of Roche Diagnostics (Hansson et al., 2018). All subjects had baseline resting-state fMRI and tau-PET whereas Aβ-PET was not available for patients with ADD. For the longitudinal part of the study, we used follow-up tau-PET scans from 422 participants. Table 1 shows the demographic and clinical characteristics of the sample.

### MRI: imaging and image processing

Structural and functional MRI was acquired using a Siemens 3T MAGNETOM Prisma scanner (Siemens Healthineers, Erlangen, Germany) with a 64-channel head coil. T1-weighted images (Magnetization Prepared, Rapid Gradient Echo, MPRAGE) were acquired with the following parameters: in-plane resolution = 1×1 mm^2^, slice thickness = 1 mm, repetition time (TR) = 1900 ms, echo time (TE) = 2.54 ms, flip-angle = 9°. Resting-state fMRI data (eyes closed) were acquired using a 3D echo-planar imaging (EPI) sequence with an in-plane resolution of 3×3 mm and slice thickness 3.6 mm; echo time = 30 ms, and flip-angle = 63°. Scan time was 7.85 minutes, with a multiband repetition time of 1020 ms, resulting in 462 frames per scan before processing and censoring. Preprocessing has briefly been described in (Berron et al., 2021). The processing was performed using a modified CPAC (Craddock et al., 2013) pipeline, building mostly on FSL (Jenkinson et al., 2012), AFNI (Cox, 1996) and ANTS (Avants et al., 2014). Skullstripping was done with in-house code using the T2 structural image as a primer and Brain Surface Extractor from BrainSuite (Shattuck and Leahy, 2002).

The fMRI preprocessing included slice-timing and motion correction. Physiological noise was regressed out using CompCor (Behzadi et al., 2007), alongside the removal of linear and quadratic trends. Additionally, regression of Friston’s 24-parameter motion correction (Friston et al., 1996), white matter and CSF signals were performed. Susceptibility distortion was corrected by unwarping the functional data using a nonlinear diffeomorphic transformation (performed with ANTS) of the mean functional image to high resolution (1×1×1 mm3) T2 structural image (Wang et al., 2017). This transformation was then applied to each volume in the fMRI time-series. Finally, a bandpass filter (0.01–0.1 Hz) was applied. For each scan, frames were censored based on DVARS for that frame lying outside above and below the third and first quartiles, respectively 1.5 × IQR (Power et al., 2012). To avoid distortion from the outlier frames, they were interpolated before bandpass filtering and then removed from the final 4D image. Finally, any participant with an average frame displacement (FD) > 0.5 mm or maximum FD > 3 mm over the entire sequence were filtered out before analysis.

### PET: imaging and image processing

All study participants underwent tau PET scans, and a subset of them as described above underwent Aβ-PET scans, all on digital GE Discovery MI scanners (General Electric Medical Systems). BioFINDER PET scanning procedures have been previously described in detail for tau-PET (Smith et al., 2020) and Aβ-PET (Palmqvist et al., 2014). Briefly, for tau PET, participants were injected with an average of 365±20 MBq [^18^F]RO948, and emission data was acquired in LIST mode between 70 and 90 minutes post-injection, adjusted for the tracer’s pharmacokinetics; for Aβ PET, an average of 185 MBq [^18^F]flutemetamol was used as tracer and emission data was acquired between 90 and 110 minutes post-injection. Lowdose CT scans were conducted before PET scans for attenuation correction. PET data was reconstructed using the VPFX-S algorithm (ordered subset expectation maximization with time-of-flight and point spread function corrections). The LIST mode data was binned into 4×5-minute time frames, and images were motion-corrected, summed, and co-registered to corresponding T1-weighted MRI images. FreeSurfer parcellation (v6; https://surfer.nmr.mgh.harvard.edu) was applied to extract regional uptake values, normalized to the mean uptake in inferior cerebellar grey matter as the reference region for [^18^F]RO948 and the whole cerebellum for [^18^F]flutemetamol.

### Subject-space cortical parcellation of PET and fMRI

We generated subject-space cortical parcellations at 10 different spatial resolutions (100 to 1000 regions, with a step of 100) defined by the Schaefer atlas, a cortical parcellation based on functional connectivity patterns and spatial contiguity. First, subject-specific cortical surfaces were derived from T1-weighted scans using FreeSurfer (FS) v6.0 (https://surfer.nmr.mgh.harvard.edu/); this procedure and all following steps are done separately for each hemisphere. Next, the cortical surface annotation file for the Schaefer atlas at each spatial resolution (defined in fsaverage space) was projected to each subject’s FS-derived surface (via FS function mri_surf2surf). Preprocessed tau-PET, Aβ-PET, and rs-fMRI (each time frame) data of each subject were coregistered to the individual’s FreeSurfer T1-weighted image (a 1 mm cubic resampled version of the original T1-weighted image on which FreeSurfer works) and subsequently projected onto the cortical surface by sampling volumetric data at the midpoint of the cortical ribbon (via FS function mri_vol2surf with the –projfrac 0.5 option). Cortical maps were then parcellated using atlas annotation files via averaging surface vertex values belonging to each annotation parcel (via FS function read_annotation, together with in-house functions). For PET data this yielded parcellated maps which were directly used for downstream analyses whereas for fMRI data regional time series were obtained that were subsequently used to construct an individual functional connectome for each atlas resolution.

### Functional connectivity estimation

For each atlas with P cortical parcels, and each subject, pairwise Pearson correlations were computed between the rs-fMRI time course of each parcel, resulting in P×P symmetric correlation matrix, representing in its columns the degree of co-fluctuation of each region with the rest of the regions. Matrix diagonals (auto-correlations) and negative values (anti-correlations) were set to zero. Remaining elements were Fisher Z-transformed (for extending dynamic range). No adhoc thresholding was applied to ensure retaining the networks connected and to prevent discarding any subtle topological information that may be deemed valuable when studying the relation between individual connectivity and tau-PET. For each atlas, a group-averaged FC was generated by averaging the FC matrices of cognitively unimpaired Aβ- individuals, an FC that served as template FC throughout our analyses.

### Null models for subject FC

To test whether the increased explained variance from adding subject-specific FC simply reflected the addition of another regressor or the capture of coarse graph features, we generated null-model FC matrices that preserved low-level network properties while randomizing higher-order topology. The first approach used a degree-preserving rewiring algorithm (Maslov and Sneppen, 2002). In this method, edges of the subject FC matrix were repeatedly rewired while maintaining each node’s degree, thereby preserving the distribution of the number of connections per region but eliminating the specific wiring pattern. This ensures that any additional variance explained cannot be attributed to differences in nodal degree alone. The second approach used a recently developed simulated annealing algorithm for weighted networks (Milisav et al., 2025). This method extends degree-preserving rewiring by additionally preserving nodal strength, i.e., the total connection weight of each region, while randomizing the arrangement of weights across edges. By controlling for both degree and strength, this null model tests whether the benefit of subject FC arises from more subtle patterns of connection placement beyond coarse local connectivity measures. Each null FC was then entered into the same regression pipeline as the empirical subject FC, allowing direct comparison of explained variance across models.

### Canonical on- and off-target tracer binding PET patterns

Building on prior work (Vogel et al., 2020), we applied two-component Gaussian mixture modelling to PET SUVR values for each cortical parcel and atlas. Models were fit to the full cohort: 805 patients (CN Aβ-, CN Aβ+, SCD Aβ+, MCI Aβ+, ADD Aβ+) for tau-PET and 422 patients (CN Aβ-, CN Aβ+, SCD Aβ+, MCI Aβ+) for Aβ-PET. Each model yielded two overlapping Gaussian curves per modality, atlas, and parcel, representing estimated off-target and on-target binding distributions; see Fig. S5 for a schematic overview of the process. While previous studies used these distributions to convert SUVRs to PET positivity probabilities (Vogel et al., 2020; Xiao et al., 2025), we instead aimed to derive canonical binding patterns that summarize cohort-level on- and off-target signals. These patterns allowed us to assess how well individual PET profiles align with canonical distributions versus more complex, connectivity-based models. The right-shifted Gaussian (higher mean) was designated as on-target binding. The means defined canonical off- and on-target values, generating two parcellated cortical maps per atlas (S6–S7, left). Standard deviations captured uncertainty, revealing low spatial variability in off-target binding but substantial variability in on-target binding, positively correlated with the mean patterns (S6–S7, right).

### Regional-level regression of individual tau-PET patterns on functional connectivity

To assess the added value of subject-specific functional connectivity (FC) in explaining individual tau-PET topographies, we applied linear regression models at the regional level (Fig. 2a). For each subject and each cortical parcellation atlas with P parcels, we predicted the subject’s tau-PET signal using the FC profile of each region as a regressor. Specifically, for a given parcel k, we regressed the subject’s tau-PET values across the remaining P*−*1 regions onto the k-th column of the FC matrix, excluding the diagonal element. This procedure was repeated for both subject-specific FC and the group-level template FC, yielding one explained variance (R^2^) value per region, per subject, and per FC modality.

To determine whether subject-specific FC provides explanatory power beyond the template FC, we fit two-predictor linear models that included both the regional FC vector from the template and a second regressor derived from either subject-specific FC, a degree-preserving null FC, or a strength-preserving null FC. Importantly, the second regressor was orthogonalized with respect to the template FC regressor using a projection-removal procedure. This step removes the component of subject FC that lies along the template FC direction, thereby isolating the unique or residual component of subject FC. In other words, it identifies the part of the subject FC that is independent of the group-level template, ensuring that both regressors explain distinct sources of variance in the tau-PET signal.

Both the template FC and the orthogonalized subject FC regressors were 2-norm normalized prior to model fitting. This normalization was essential to ensure that the regression coefficients (*β*_1_ for template FC and *β*_2_ for subject FC) are comparable and interpretable, since otherwise the scale differences between regressors would bias coefficient estimates. The corrected R^2^ values from these models provided a matched distribution of variance explained across regions, subjects, and FC types, enabling fair comparison of predictive performance across models.

### Whole-Brain regression of individual tau-PET patterns on functional connectivity

To extend the analysis beyond region-wise prediction and evaluate the global relationship between FC and tau pathology, we fit linear models using the full FC profiles of each subject (Fig. 1a). For each FC modality, i.e., subject FC, template FC, and hybrid FC, all regional connectivity vectors within the left hemisphere were used simultaneously to predict the subject’s tau-PET distribution in the same hemisphere. Self-connections were excluded, and each FC regressor vector was 2-norm normalized prior to inclusion.

Because the number of predictors equals the number of observations (i.e., number of cortical parcels), we performed the modelling separately for each hemisphere to avoid overfitting, then averaged the R^2^ values from the left and right hemispheres for each subject and FC type. This approach yielded one R^2^ value per subject per FC model, summarizing the extent to which each FC modality explained individual variability in tau-PET across the cortex. This analysis provides a complementary, system-level perspective to the regional models and enables direct comparison across different FC representations.

### Hybrid functional connectivity profiles

To enable joint modelling of subject-specific and group-level FC effects in a tractable and principled way, we constructed hybrid functional connectivity (hybrid FC) profiles for use in the whole-brain modelling analysis described above. While the regional regression models allowed us to estimate the separate contributions of subject FC and template FC, including both as predictors in full-brain models would double the number of regressors (i.e., 2P for P cortical parcels), which is infeasible given the number of observations. The hybrid FC approach addresses this by combining both FC sources into a single profile, preserving dimensionality while integrating both shared and individual-specific connectivity information.

In addition to its practical advantages, the hybrid FC method addresses concerns around the reliability of subject FC in clinical populations. Resting-state fMRI data in clinical cohorts often suffer from limited scan time, motion artifacts, and low signal-to-noise ratio, making individualized FC estimates noisy and unstable. As a result, prior studies frequently rely on group-averaged (template) FC profiles to study tau-PET patterns. To mitigate noise, some approaches apply ad hoc thresholding to subject FC matrices to remove weak connections; however, this introduces arbitrariness and may compromise network integrity.

Our hybrid FC strategy avoids these limitations by applying no thresholding to the FC matrices and instead leveraging a data-driven denoising mechanism. Specifically, for each region and subject, we constructed a linear combination of the subject’s original (non-orthogonalized) FC vector and the template FC vector, using region-specific effect sizes (*β*_1_ and *β*_2_) estimated from the two-predictor regional regression models. Both input vectors were 2-norm normalized before weighting, and the resulting hybrid FC vector was normalized again after combination.

This procedure yields a single, integrated FC profile per region that reflects the weighted contributions of both normative and individual-specific features. The template FC captures stable, canonical connectivity patterns, while the subject FC retains personalized deviations. By tuning their combination based on predictive performance, the hybrid FC profile effectively denoises subject-level signal while still preserving meaningful idiosyncrasies. This approach allows us to model the relationship between FC and tau pathology using the same number of predictors as for the individual FC types, enabling fair and interpretable comparisons across subject FC, template FC, and hybrid FC models in whole-brain analyses. Because hybrid FC replaces the original subject and template regressors with weighted combinations derived from regional fits, improvement at the whole-brain level is not mathematically guaranteed for every individual; occasional parity or small decrements are expected.

### Non-parametric estimation of effect sizes, confidence intervals, and p-values

To assess the difference between the performance of hybrid FC and template FC across spatial scales and clinical groups, we used a non-parametric resampling-based procedure to estimate effect sizes (Cohen’s *d*), 95% confidence intervals (CIs), and permutation-based p-values. All comparisons were conducted on paired data, comparing model performance for the same set of individuals. Cohen’s *d* was computed as the mean difference in explained variance between models, divided by the standard deviation of the within-subject differences. Confidence intervals for the effect size were obtained via bootstrapping with 10,000 iterations. On each iteration, a bootstrap sample was drawn with replacement from the vector of within-subject differences, and Cohen’s *d* was recomputed. The 95% CI was defined as the 2.5th and 97.5th percentiles of the resulting distribution of bootstrap estimates. Permutation-based p-values were computed using a sign-flipping approach: for each of 10,000 iterations, the signs of the within-subject differences were randomly flipped to simulate the null distribution. The two-tailed p-value was then defined as the proportion of permutations where the absolute mean difference was greater than or equal to the observed value. This non-parametric approach avoids assumptions of normality, controls for subject-level variability, and yields robust estimates of effect size and statistical significance across spatial scales. Because effect sizes and their confidence intervals are estimated via resampling, the approach naturally accommodates differences in group sample sizes, allowing for consistent interpretation of Cohen’s *d* across clinical groups.

For between-group comparisons, where performance was compared across independent subject groups (e.g., CN Aβ- vs. CN Aβ+), we applied the same resampling-based framework adapted for unpaired data. Cohen’s *d* was computed as the difference in group means divided by the pooled standard deviation. Bootstrapped confidence intervals for the effect size were generated using 10,000 iterations, resampling with replacement separately within each group. Permutation-based p-values were obtained by permuting group labels and recomputing the mean difference on each iteration to construct a null distribution. These procedures allow valid statistical inference without assumptions of equal variance or normality, and remain robust even in the presence of unequal group sizes.

### Association between baseline pathology and explained variance of baseline and follow-up tau-PET

To test whether baseline pathology influenced the predictive performance of hybrid FC, we fit linear models relating baseline Aβ-PET, early tau-PET (Braak I–IV), or late tau-PET (Braak V–VI) to the explained variance of tau-PET at baseline and follow-up (Fig. 5c–e), using hybrid FC derived from each individual’s baseline resting-state fMRI. The analysis aimed (i) to determine how well baseline-derived hybrid FC explains tau-PET patterns at follow-up and (ii) to assess how explained variance in baseline and follow-up tau-PET relates to baseline pathological measures. All predictors and outcomes were z-scored to obtain standardized effect sizes, and age and sex were included as covariates. To evaluate robustness, the analysis was repeated across spatial scales.

## Supplementary Material

1

## Figures and Tables

**Table 1.** Demographics and clinical characteristics of the sample. Aβ: β-amyloid; Aβ status (positive: Aβ+, negative: Aβ-) was determined based on CSF Aβ42/Aβ40 ratio. Data are presented as mean±s.d. unless specified otherwise. CN: Cognitively normal; SCD: Subjective cognitive decline; MCI: mild cognitive impairment; ADD: Alzheimer’s disease dementia; MMSE: mini mental state examination; *APOE* ε4: apolipoprotein E genotype carrying at least one ε4 allele.

	CN Aβ− (n=309)	CN Aβ+ (n=89)	SCD Aβ+ (n=77)	MCI Aβ+ (n=156)	ADD Aβ+ (n=174)	Overall (n=805)
**Age (years)**	67.27 ± 10.95	76.29 ± 8.14	70.55 ± 6.96	72.74 ± 6.82	72.98 ± 7.70	70.88 ± 9.45
**Sex, female n (%F)**	188 (61)	49 (55)	37 (48)	69 (44)	99 (57)	442 (55)
**Education (years)**	12.67 ± 3.41	12.28 ± 3.92	13.09 ± 3.95	13.16 ± 4.48	12.08 ± 4.05	12.64 ± 3.89
***APOE* status available n**	225	45	75	153	172	670
***APOE*** ε**4 carriers n (% of available n)**	98 (43.0)	34 (75.6)	54 (72.0)	117 (76.6)	122 (70.9)	425 (63.43)
**MMSE**	29.05 ± 1.05	28.52 ± 1.32	28.55 ± 1.50	27.06 ± 1.89	21.11 ± 4.02	26.85 ± 3.81
**tau-PET SUVR (RO948), Braak Stages I-IV** ^a^	1.14 ± 0.08	1.24 ± 0.18	1.33 ± 0.29	1.50 ± 0.43	2.12 ± 0.63	1.45 ± 0.52
**tau-PET SUVR (RO948), Braak Stages V-VI** ^a^	1.04 ± 0.07	1.06 ± 0.11	1.09 ± 0.13	1.16 ± 0.20	1.52 ± 0.40	1.17 ± 0.29
**follow-up tau-PET, available n (female n)**	175 (101)	46 (24)	54 (24)	73 (26)	74 (44)	422 (219)
**tau-PET follow-up time (years)**	1.91 ± 0.53	1.88 ± 0.63	1.75 ± 0.33	1.74 ± 0.35	1.60 ± 0.41	1.80 ± 0.48
**A**β**-PET SUVR**^b^ **(flutemetamol), meta ROI**	0.92 ± 0.04	1.34 ± 0.22	1.43 ± 0.23	1.52 ± 0.27	Not available^c^	1.20 ± 0.33

a Braak staging meta-ROI (Braak and Braak, 1991) was constructed using regions defined by (Cho et al., 2016), based on FreeSurfer labels.

b There are 4, 0, 0, 0, 174 missing values in the respective categories; value defined as the average SUVR of a global neocortical ROI (Ossenkoppele et al., 2022), including prefrontal, lateral temporal, parietal, anterior cingulate and posterior cingulate/praecuneus.

c Dementia patients did not undergo Aβ-PET.
