# Supplementary material for: Patient-specific functional brain architecture explains cortical patterns of tau PET in Alzheimer’s disease
Source: bioRxiv. 2025 Oct 14:2025.10.02.679969. Preprint. [Version 2] doi: 10.1101/2025.10.02.679969 (PMC12621874; doi:10.1101/2025.10.02.679969)
Supplement: 1 [file NIHPP2025.10.02.679969V2-supplement-1.pdf]

bioRxiv preprint doi: https://doi.org/10.1101/2025.10.02.679969; this version posted October 14, 2025. The copyright holder for this preprint (which was not certified by peer review) is the author/funder, who has granted bioRxiv a license to display the preprint in perpetuity. It is made available under aCC-BY 4.0 International license.

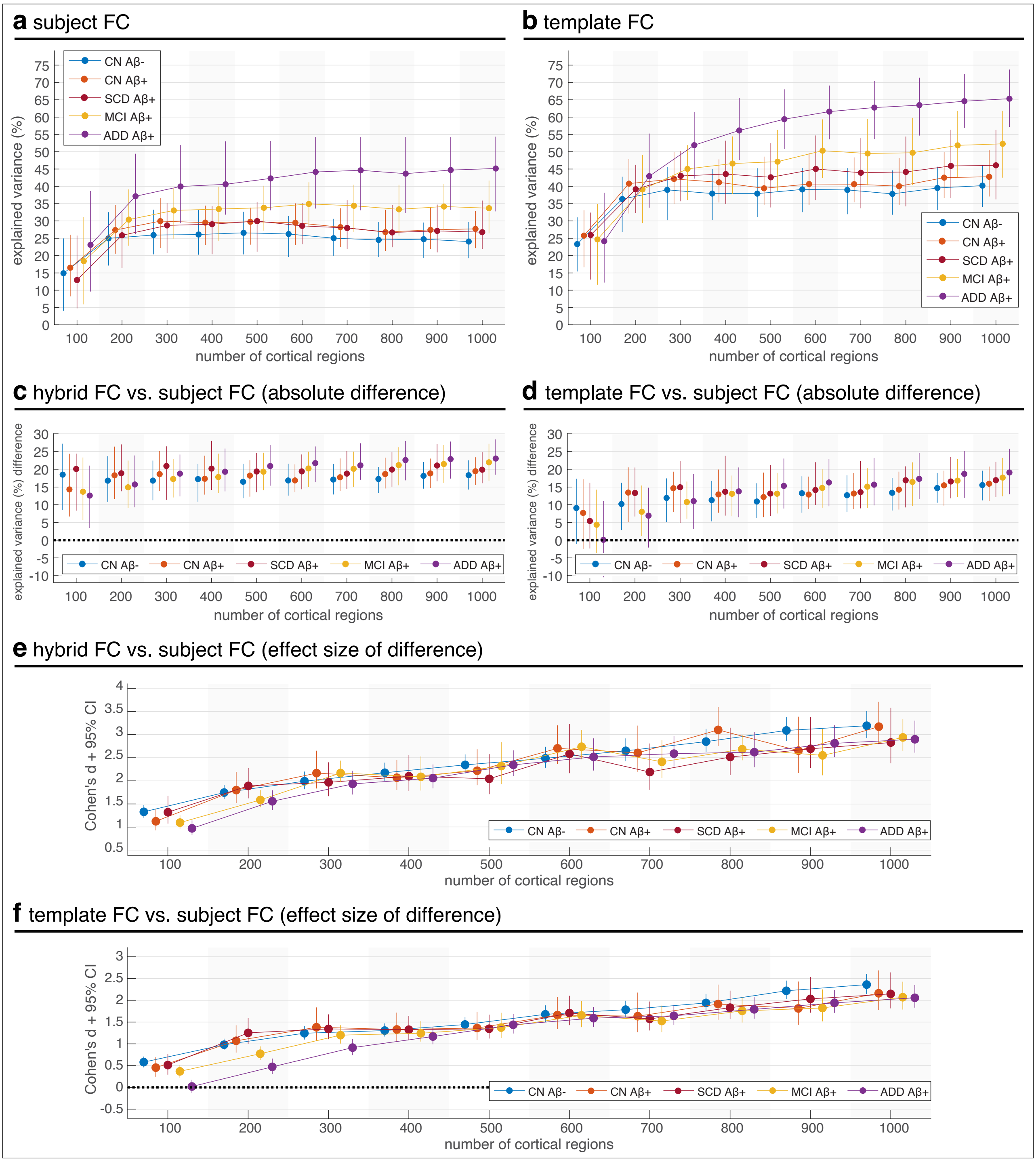

Fig. S1 | Hybrid FC consistently outperforms subject FC and template FC in explaining individual tau-PET patterns across spatial scales and clinical stages. (a) Explained variance (R^2) from subject FC increases modestly with spatial granularity, peaking in ADD patients. (b) Template FC explains more variance than subject FC across groups, especially at finer scales and in ADD, echoing Fig. 2c (top). (c) Hybrid FC outperforms subject FC, with the largest gains in MCI and ADD, particularly at finer parcellations. (d) These gains exceed those from template FC vs. subject FC across groups and scales. (e–f) Corresponding effect sizes (Cohen's *d*) for panels (c) and (d), showing that hybrid FC's advantage over subject FC grows with granularity and surpasses that of template FC. These results support Fig. 2, showing that hybrid FC (a combination of subject- and template FC) outperforms both components alone. While subject FC explains little variance early in disease, hybrid FC significantly improves performance, especially in MCI and ADD, and remains robust across scales. The superior performance of template FC at high resolutions (panel b vs. a) explains hybrid FC's increasing advantage with granularity (panel c). Overall, hybrid FC better captures tau-PET variability across disease stages and spatial resolutions. In panels a–d, whiskers show 25th–75th percentiles; markers indicate median. In panels e–f, whiskers denote 95% CI around Cohen's *d*; markers indicate point estimates.

bioRxiv preprint doi: https://doi.org/10.1101/2025.10.02.679969; this version posted October 14, 2025. The copyright holder for this preprint (which was not certified by peer review) is the author/funder, who has granted bioRxiv a license to display the preprint in perpetuity. It is made available under aCC-BY 4.0 International license.

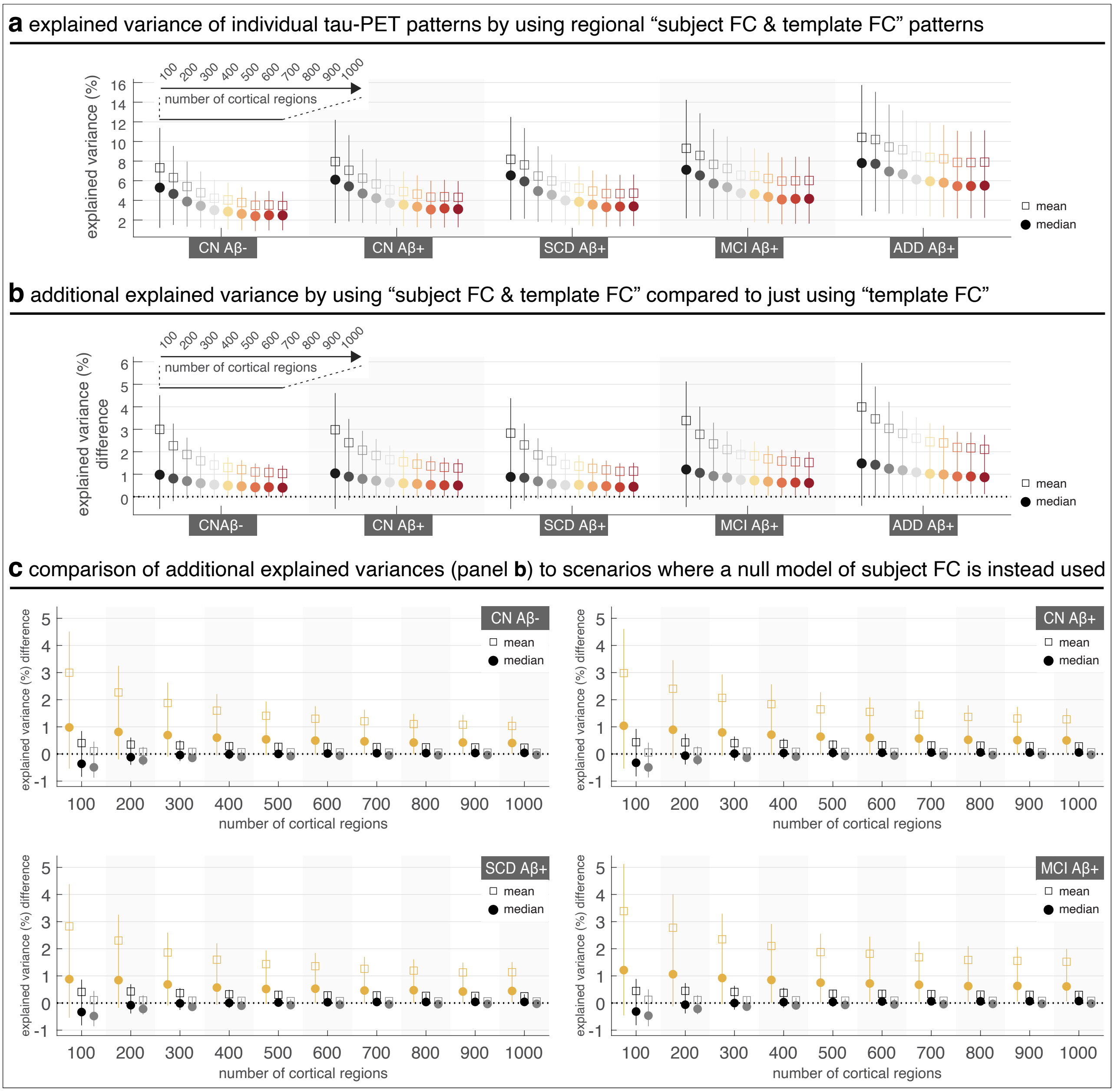

Fig. S2 | Explained variance of individual tau-PET by using both regional subject FC and template FC, across spatial scales and across groups. (a)-(b) Overall explained variance (a) and the additional explained variance compared to using just template FC (b) is greater at later stages and decreases as a function of spatial granularity of the design (i.e., cortical area covered by regions decreased). It is, however, important to note that as the number of regions in the atlases increases, FC of each region entails information of a smaller fraction of the entire FC, thus, the explained variance by regional FC decreases. (c) Additional explained variance from including subject FC, compared to two null models where subject FC was rewired to preserve either nodal degree (Maslov and Sneppen, 2002) or both nodal degree and strength (Milisav et al., 2025). Results for group ADD are shown in Fig. 2c. In all panels, whiskers represent the 25th to 75th percentiles; markers indicate the median, across regions and subjects in each group.

bioRxiv preprint doi: https://doi.org/10.1101/2025.10.02.679969; this version posted October 14, 2025. The copyright holder for this preprint (which was not certified by peer review) is the author/funder, who has granted bioRxiv a license to display the preprint in perpetuity. It is made available under aCC-BY 4.0 International license.

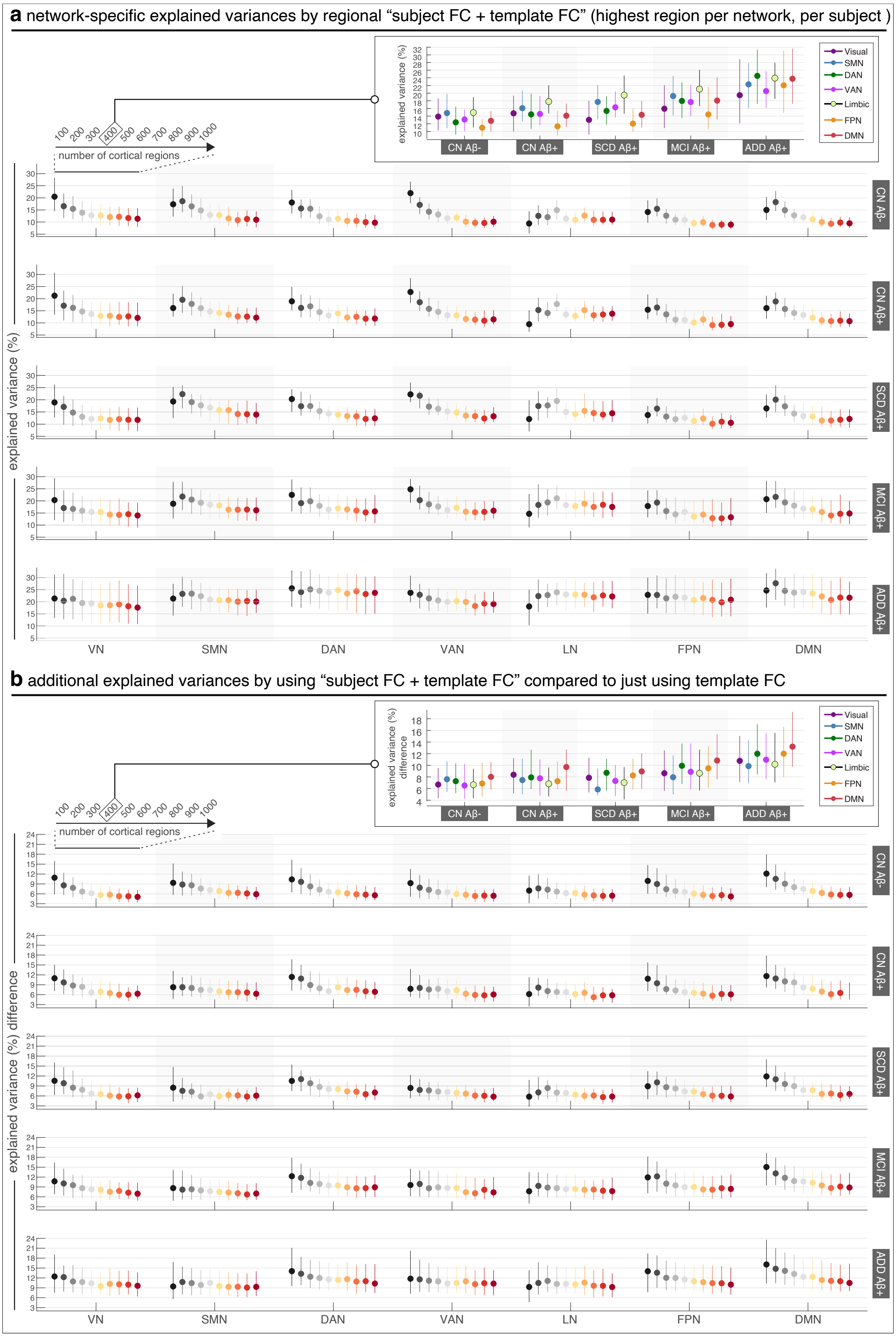

Fig. S3 | Network-specific model fits across spatial scales using regional FC. (a) Explained variance of individual tau-PET patterns using regional "subject FC & template FC". For each subject and network, the region with the highest explained variance was selected. Performance varied across networks and disease stages, with the Default Mode (DMN) and Frontoparietal (FPN) networks generally yielding the strongest fits, especially in symptomatic Aβ+ groups. The limbic network also showed notable contributions in earlier Aβ+ groups, though its performance was more sensitive to spatial resolution, peaking at intermediate atlas sizes (400 regions). (b) Additional explained variance gained by including both subject and template FC compared to template FC alone. Gains were most consistent in the DMN and FPN, while other networks (e.g., limbic and visual) showed scale- and group-dependent improvements. Together, these results highlight that individual FC contributes explanatory power beyond group-level FC, and that the most informative regions for capturing tau variability often lie within networks previously implicated in AD progression. Whiskers represent the 25th to 75th percentiles across subjects; markers indicate the median.

bioRxiv preprint doi: https://doi.org/10.1101/2025.10.02.679969; this version posted October 14, 2025. The copyright holder for this preprint (which was not certified by peer review) is the author/funder, who has granted bioRxiv a license to display the preprint in perpetuity. It is made available under aCC-BY 4.0 International license.

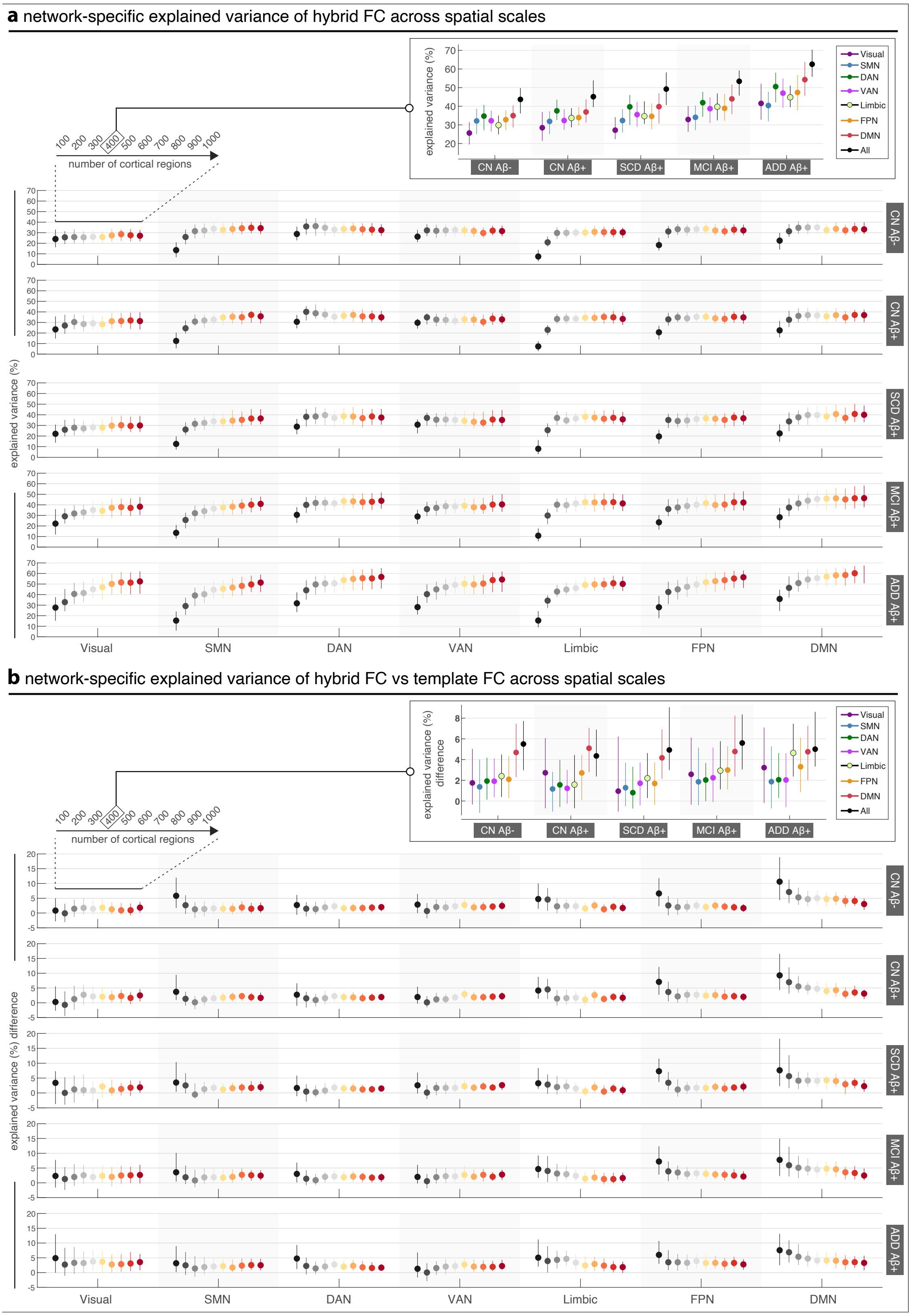

Fig. S4 | Network-specific model fits across spatial scales using regions spanning entire networks. (a) Explained variance of individual tau-PET patterns using hybrid FC, fit separately within each canonical network, across spatial scales and clinical groups. For each network, models were fit using the regional FC profiles of all regions within the network. The DMN and FPN consistently showed the highest explained variance, particularly in MCI Aβ+ and ADD groups, whereas the limbic network was stronger in earlier Aβ+ groups but plateaued or declined at finer parcellations. Visual and limbic networks also showed sharper declines in performance at higher resolutions, in contrast to the DMN and FPN, which remained stable. (b) Additional explained variance of hybrid FC relative to template FC, computed separately for each network, spatial scale, and group. Gains in the DMN and FPN were robust across scales, while benefits in the limbic and visual networks peaked at intermediate resolutions (400-600 regions). These findings emphasize that the contribution of individual FC varies by both network and disease stage, with the DMN and FPN most consistently capturing tau-related variability. Whiskers represent the 25th to 75th percentiles across subjects; markers indicate the median.

bioRxiv preprint doi: https://doi.org/10.1101/2025.10.02.679969; this version posted October 14, 2025. The copyright holder for this preprint (which was not certified by peer review) is the author/funder, who has granted bioRxiv a license to display the preprint in perpetuity. It is made available under aCC-BY 4.0 International license.

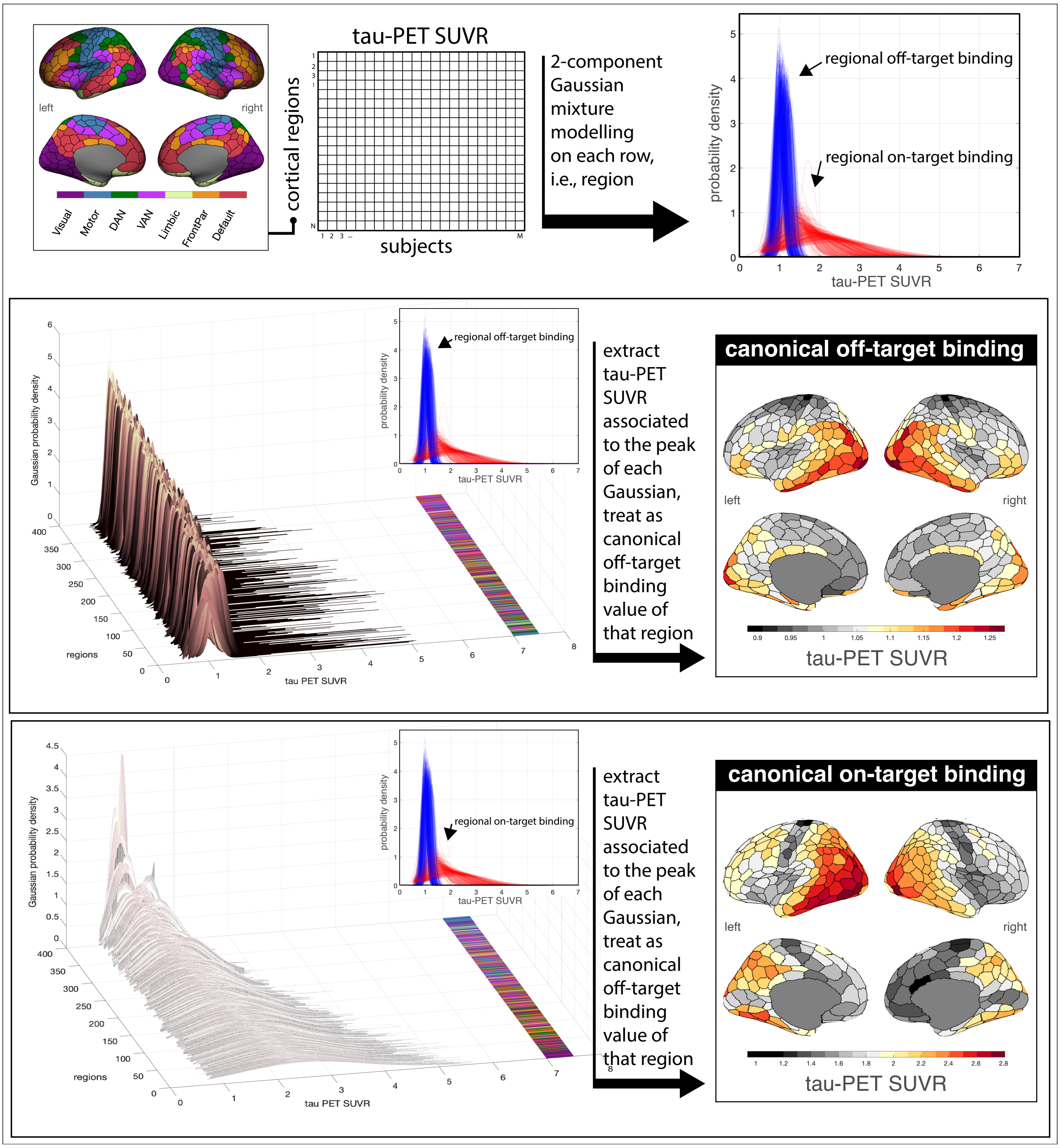

Fig. S5 | Derivation of canonical PET patterns. For each cortical region, a two-component Gaussian mixture model was fitted on tau-PET SUVR values of that region in the cohort (Vogel et al., 2020). The mean of left- and right-hand Gaussian are treated as the canonical off- and on-target tau-PET value of that region, respectively; the standard deviation of each Gaussian can also be used to reflect the uncertainty of each canonical estimate. This analysis results in an off- and on-target binding tau-PET spatial map defined at the resolution of the atlas used, with the resulting spatial map for each atlas being shown in Fig. S6 and Fig. S7, respectively.

bioRxiv preprint doi: https://doi.org/10.1101/2025.10.02.679969; this version posted October 14, 2025. The copyright holder for this preprint (which was not certified by peer review) is the author/funder, who has granted bioRxiv a license to display the preprint in perpetuity. It is made available under aCC-BY 4.0 International license.

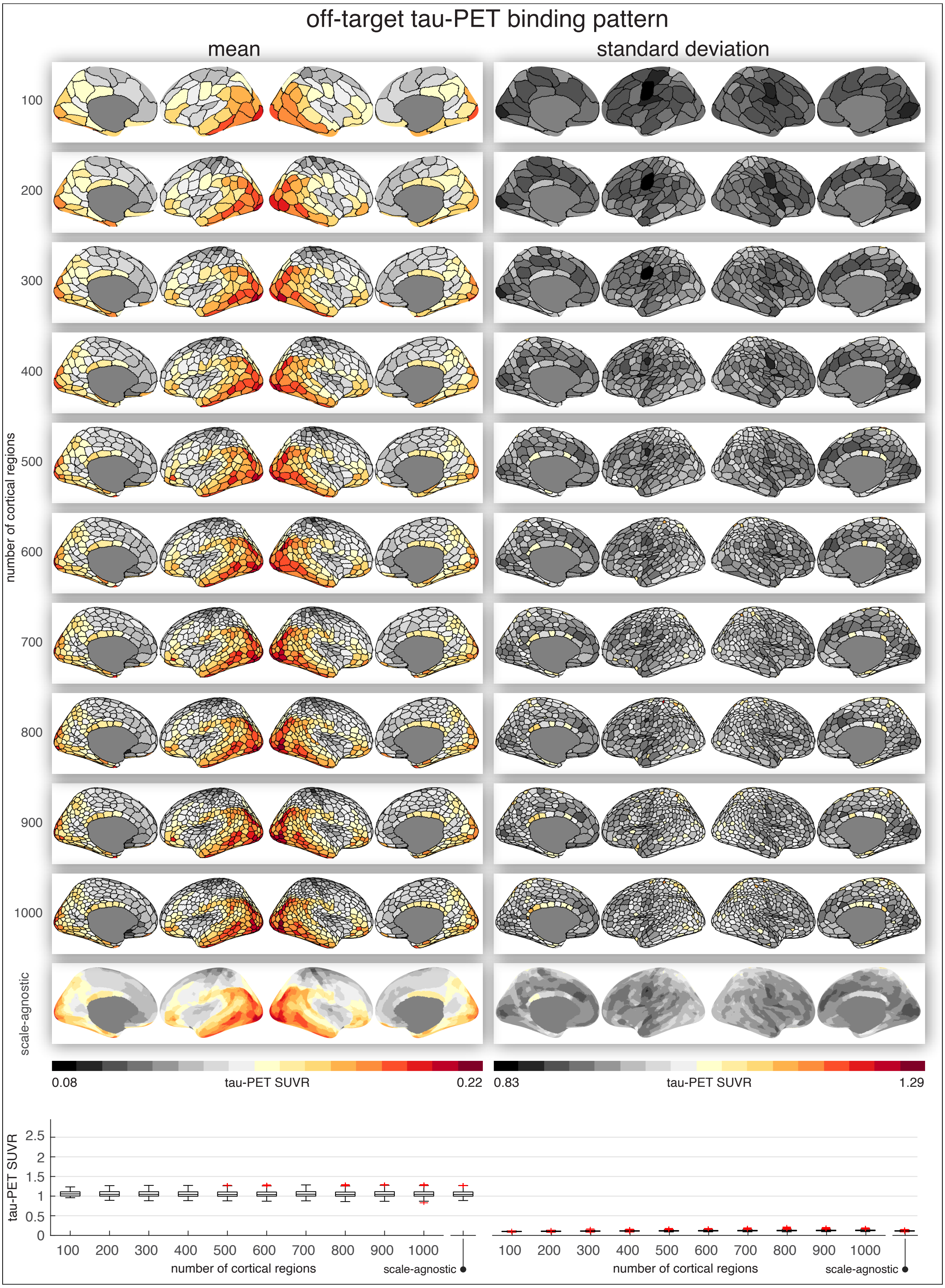

Fig. S6 | Canonical off-target binding tau-PET patterns across spatial scales. A unit set of off-target tau-PET binding value is obtained for each region defined by an atlas at a given resolution, resulting in a spatial pattern at the resolution of the atlas (see the first 10 rows of the first column); the second column shows the standard deviation of the estimated off-target binding values for each region. The last row in the surface plots show the scale-average off-target binding tau-PET maps and its standard deviation, obtained by averaging the 10 atlas-resolution maps. The bottom panel compares boxplots of the distribution of values across all the displayed surface maps using a fixed y-axis range.

bioRxiv preprint doi: https://doi.org/10.1101/2025.10.02.679969; this version posted October 14, 2025. The copyright holder for this preprint (which was not certified by peer review) is the author/funder, who has granted bioRxiv a license to display the preprint in perpetuity. It is made available under aCC-BY 4.0 International license.

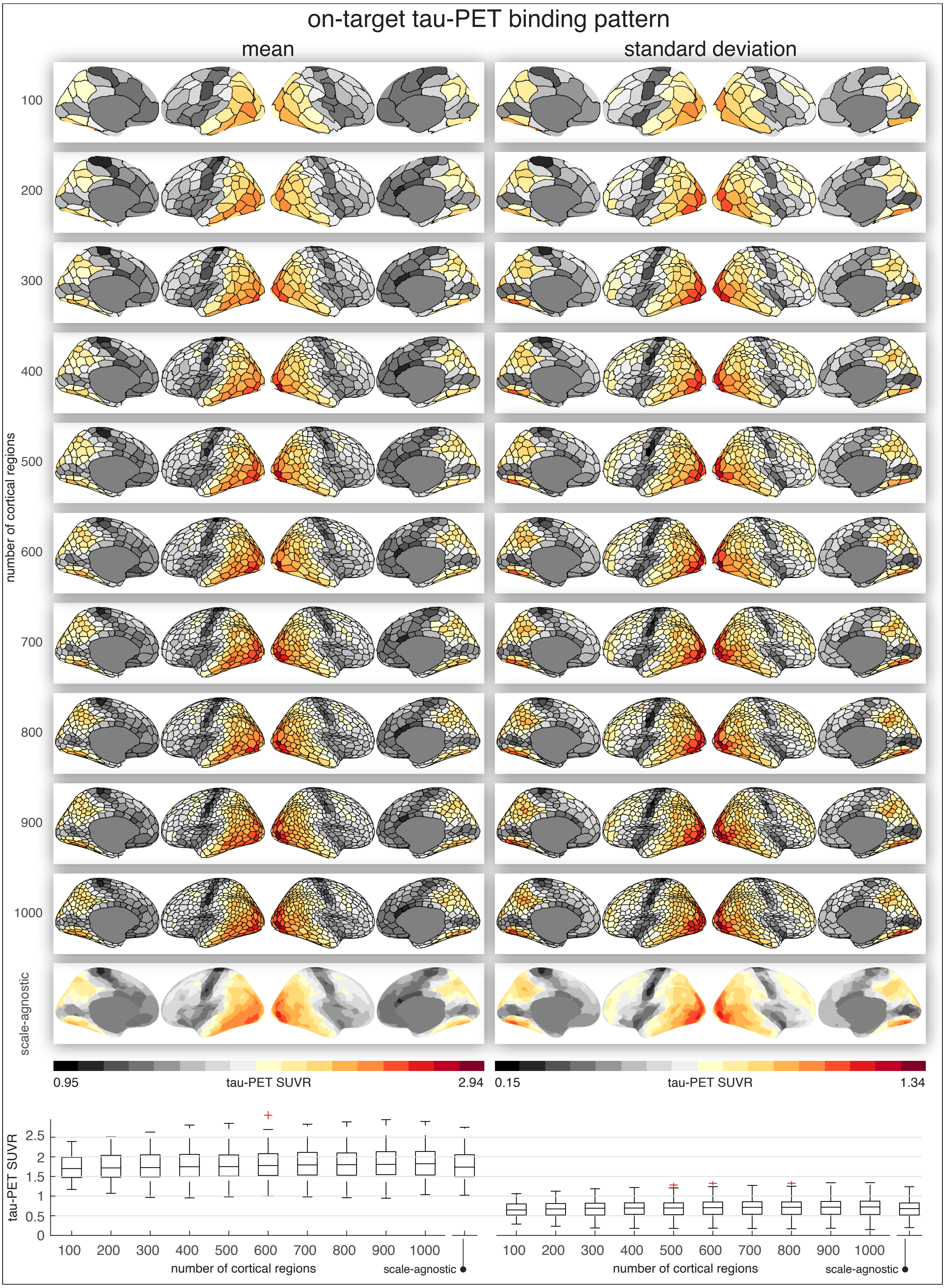

Fig. S7 | Canonical on-target binding tau-PET patterns across spatial scales. A unit set of on-target tau-PET binding value is obtained for each region defined by an atlas at a given resolution, resulting in a spatial pattern at the resolution of the atlas (see the first 10 rows of the first column); the second column shows the standard deviation of the estimated on-target binding values for each region. The last row in the surface plots show the scale-average on-target binding tau-PET maps and its standard deviation, obtained by averaging the 10 atlas-resolution maps. The bottom panel compares boxplots of the distribution of values across all the displayed surface maps using a fixed y-axis range.

bioRxiv preprint doi: https://doi.org/10.1101/2025.10.02.679969; this version posted October 14, 2025. The copyright holder for this preprint (which was not certified by peer review) is the author/funder, who has granted bioRxiv a license to display the preprint in perpetuity. It is made available under aCC-BY 4.0 International license.

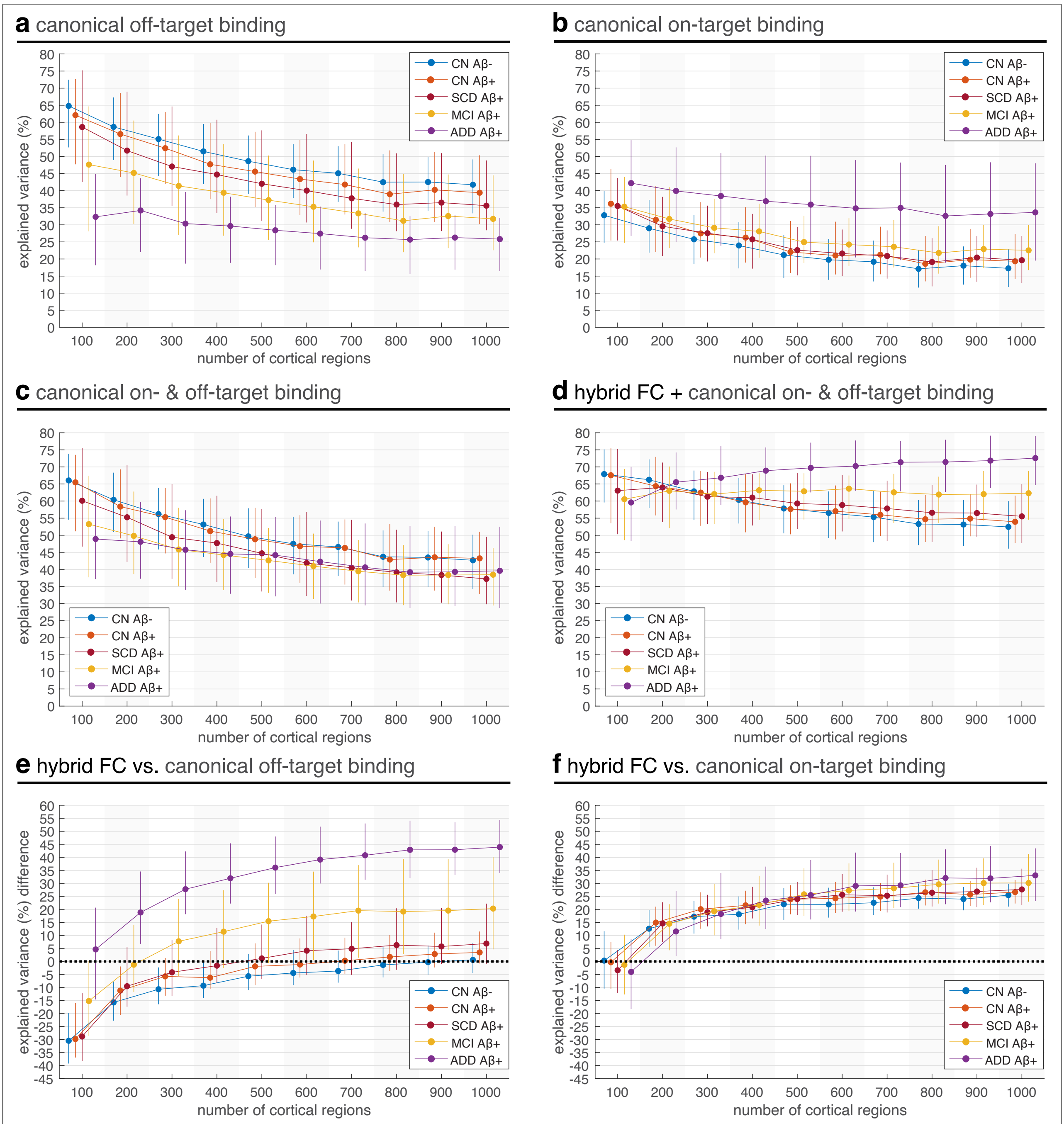

Fig. S8 | Explained variance of individual tau-PET using canonical tau-PET regressors: (a) canonical off-target binding tau-PET pattern, (b) canonical on-target binding tau-PET pattern, (c) canonical off- and on-target tau-PET binding patterns, and (d) hybrid FC and canonical off- and on-target binding patterns, across scales and across groups. Difference in explained variance of individual tau-PET patterns using hybrid FC compared to using (e) canonical off-target tau-PET binding pattern and (f) canonical on-target tau-PET binding pattern. In (a)-(f), explained variances represent "corrected" R^2 to enable unbiased comparison of explained variance using models with different numbers of regressors. In all panels, whiskers represent the 25th to 75th percentiles across subjects; markers indicate the median.

bioRxiv preprint doi: https://doi.org/10.1101/2025.10.02.679969; this version posted October 14, 2025. The copyright holder for this preprint (which was not certified by peer review) is the author/funder, who has granted bioRxiv a license to display the preprint in perpetuity. It is made available under aCC-BY 4.0 International license.

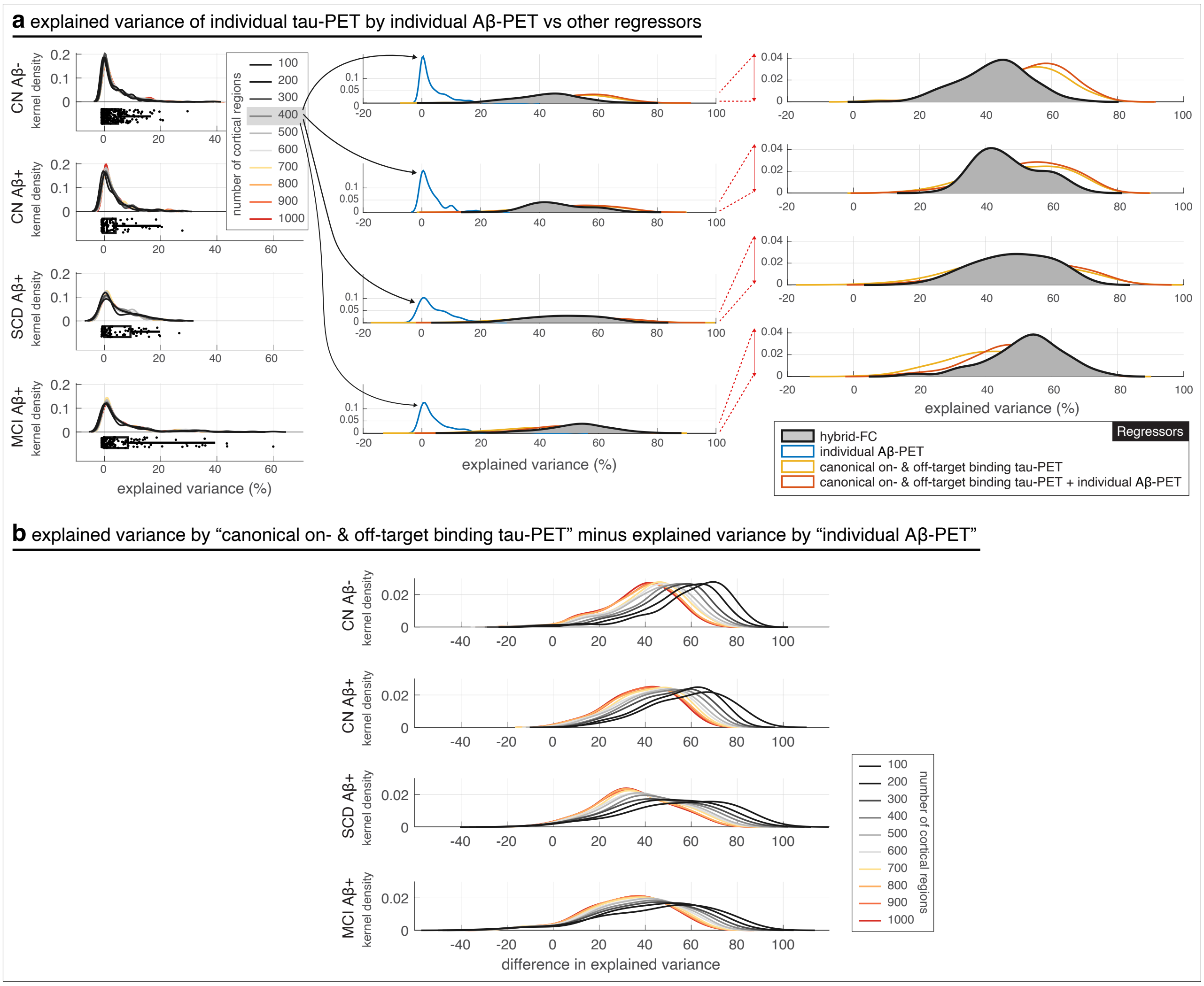

Fig. S9 | Comparison of performance of individual Aβ-PET patterns in explaining individual tau-PET patterns relative to the performance of canonical tau-PET patterns and individual hybrid FC. Patients with ADD are not included in results in this figure since they did not have an Aβ-PET scan. (a) Explained variances of individual tau-PET patterns using individual Aβ-PET are compared to other regressors; performance of individual Aβ-PET across spatial scales is shown on the left and on the right its performance is compared against other regressors for cortical parcellation with 400 regions. Individual Aβ-PET on its own minimally explains the spatial profile of individual tau-PET patterns, across spatial scales. Furthermore, when combined with canonical tau-PET patterns, the explained variance only minimally increases in CN individuals. In patients with MCI, the increase is slightly larger, yet, the performance remains below the performance of hybrid FC in explaining individual tau-PET. (b) The difference in explained variance of individual tau-PET by canonical-tau-PET patterns relative to individual Aβ-PET patterns is shown across spatial scales. The difference in performance decreases as the spatial resolution increases across the three groups. The differences in explained variances are quite in par across the three groups at high spatial resolutions whereas it is slightly lower at low spatial resolution in patients with MCI.

bioRxiv preprint doi: https://doi.org/10.1101/2025.10.02.679969; this version posted October 14, 2025. The copyright holder for this preprint (which was not certified by peer review) is the author/funder, who has granted bioRxiv a license to display the preprint in perpetuity. It is made available under aCC-BY 4.0 International license.

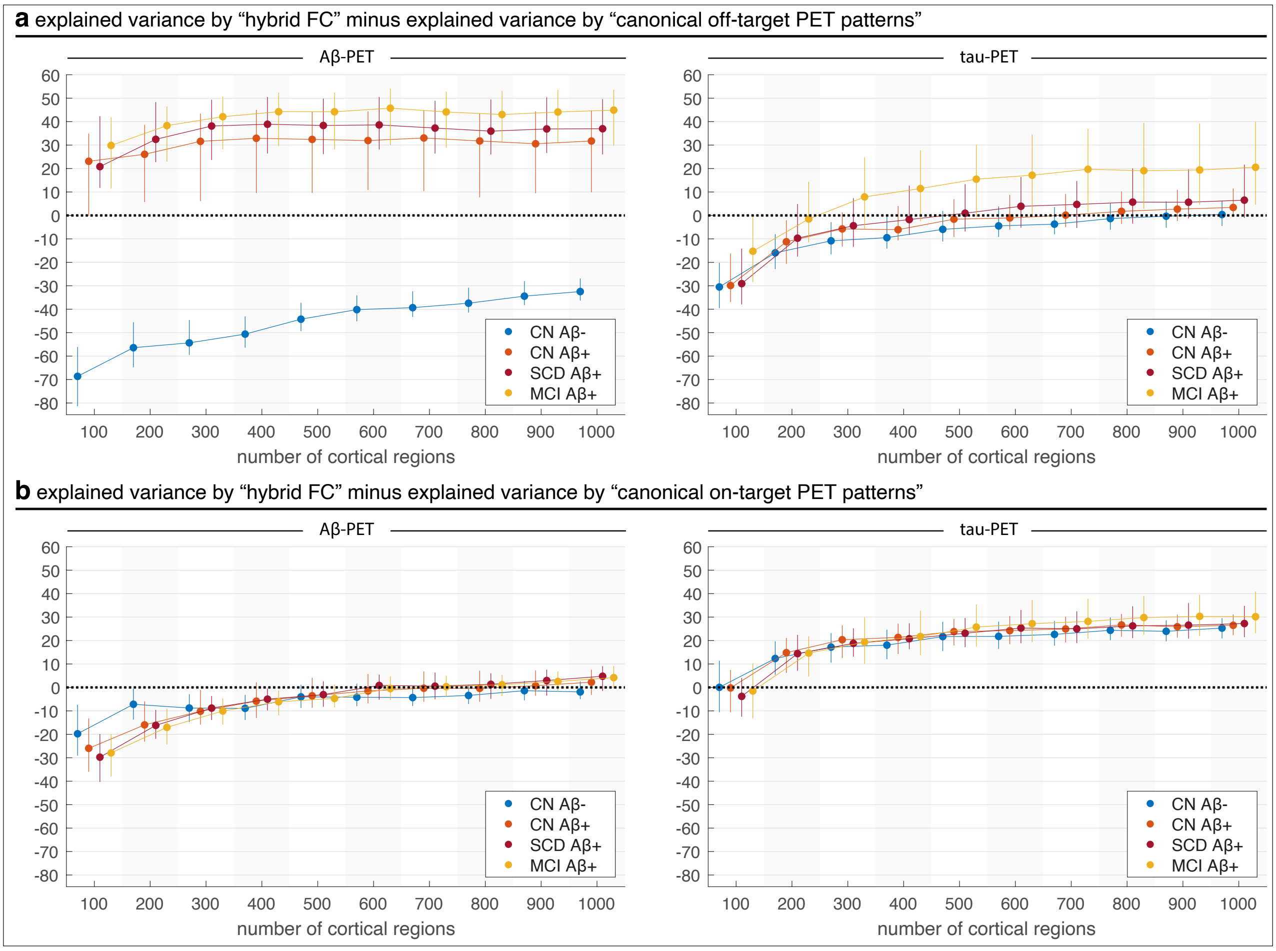

Fig. S10 | Comparison of differences in explained variance between hybrid FC and canonical PET pattern models for individual tau-PET and Aβ-PET, across spatial scales. (a) Hybrid FC vs. canonical off-target patterns: Aβ-PET is better explained by canonical patterns in CN Aβ+ and MCI, while hybrid FC outperforms for tau-PET across all groups. (b) Hybrid FC vs. canonical on-target patterns: Similar Aβ-PET trend as in (a), with narrowing differences at finer scales; tau-PET remains better captured by hybrid FC. These results, together with those shown in Fig. 4b, underscore a modality-specific dissociation: Aβ-PET is better explained by canonical spatial patterns, whereas tau-PET is more accurately predicted by functional connectivity-based models, particularly at finer spatial resolutions. In all panels, whiskers represent the 25th to 75th percentiles; markers indicate the median, across subjects in each group and at each scale.

bioRxiv preprint doi: https://doi.org/10.1101/2025.10.02.679969; this version posted October 14, 2025. The copyright holder for this preprint (which was not certified by peer review) is the author/funder, who has granted bioRxiv a license to display the preprint in perpetuity. It is made available under aCC-BY 4.0 International license.

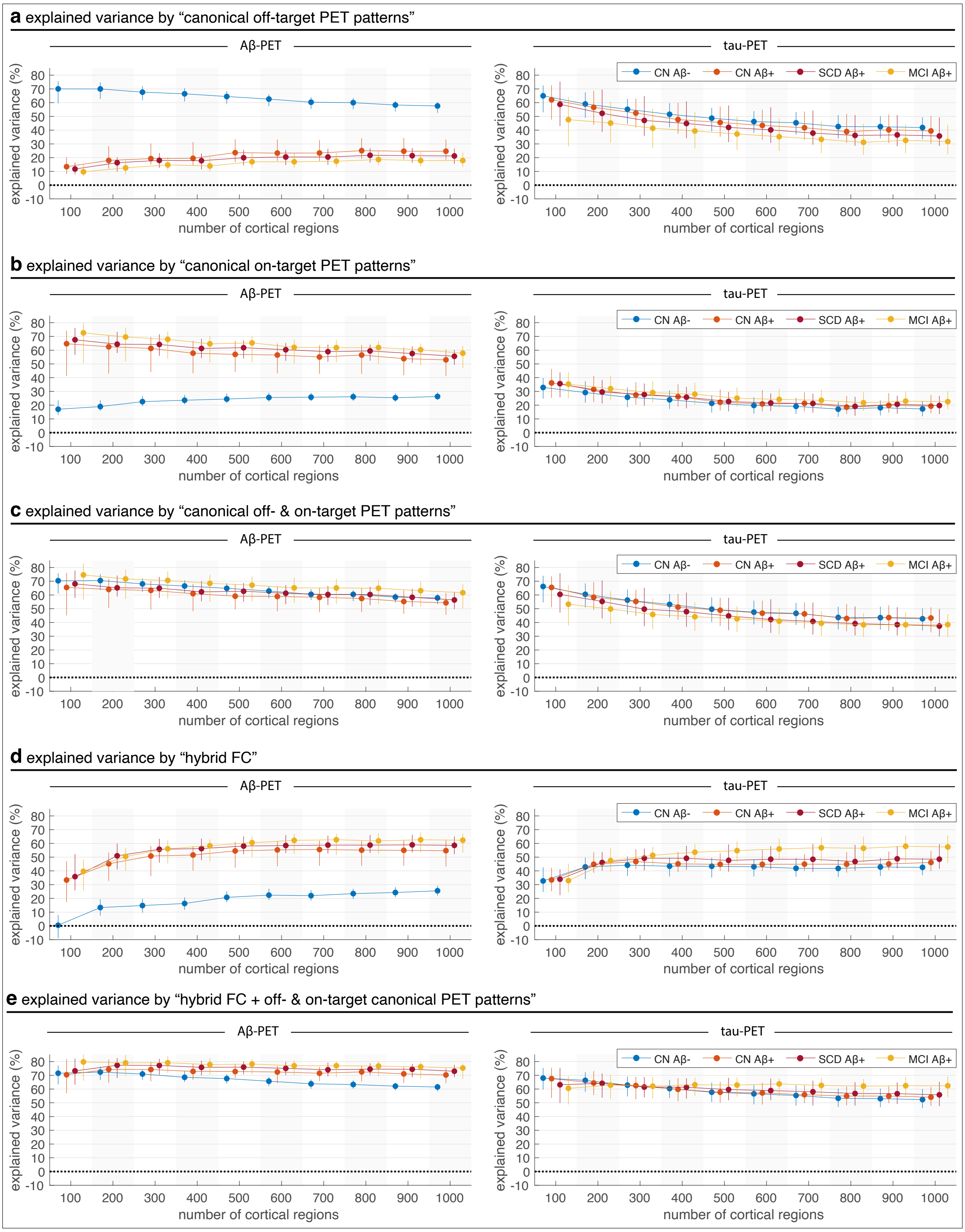

Fig. S11 | Explained variance of Aβ-PET and tau-PET patterns by different regressors across spatial scales. (a) Canonical off-target PET patterns, (b) canonical on-target PET patterns, (c) combined canonical off- & on-target PET patterns, (d) hybrid functional connectivity (FC), and (e) hybrid FC + canonical PET patterns. Canonical Aβ-PET patterns explain high Aβ-PET variance in Aβ+ groups with stable performance across scales. Tau-PET variance explained by canonical models declines with resolution and remains lower overall. Hybrid FC better explains tau-PET (especially in Aβ+ groups) than Aβ-PET, with improvements at finer scales. For Aβ-PET, hybrid FC adds little beyond canonical models. For tau-PET, combining hybrid FC with canonical PET patterns improves performance at higher resolutions. Results highlight modality-specific patterns: Aβ-PET aligns with predefined spatial maps; tau-PET is better captured by FC-based models. Whiskers: 25th–75th percentiles; markers: median across subjects.

bioRxiv preprint doi: https://doi.org/10.1101/2025.10.02.679969; this version posted October 14, 2025. The copyright holder for this preprint (which was not certified by peer review) is the author/funder, who has granted bioRxiv a license to display the preprint in perpetuity. It is made available under aCC-BY 4.0 International license.

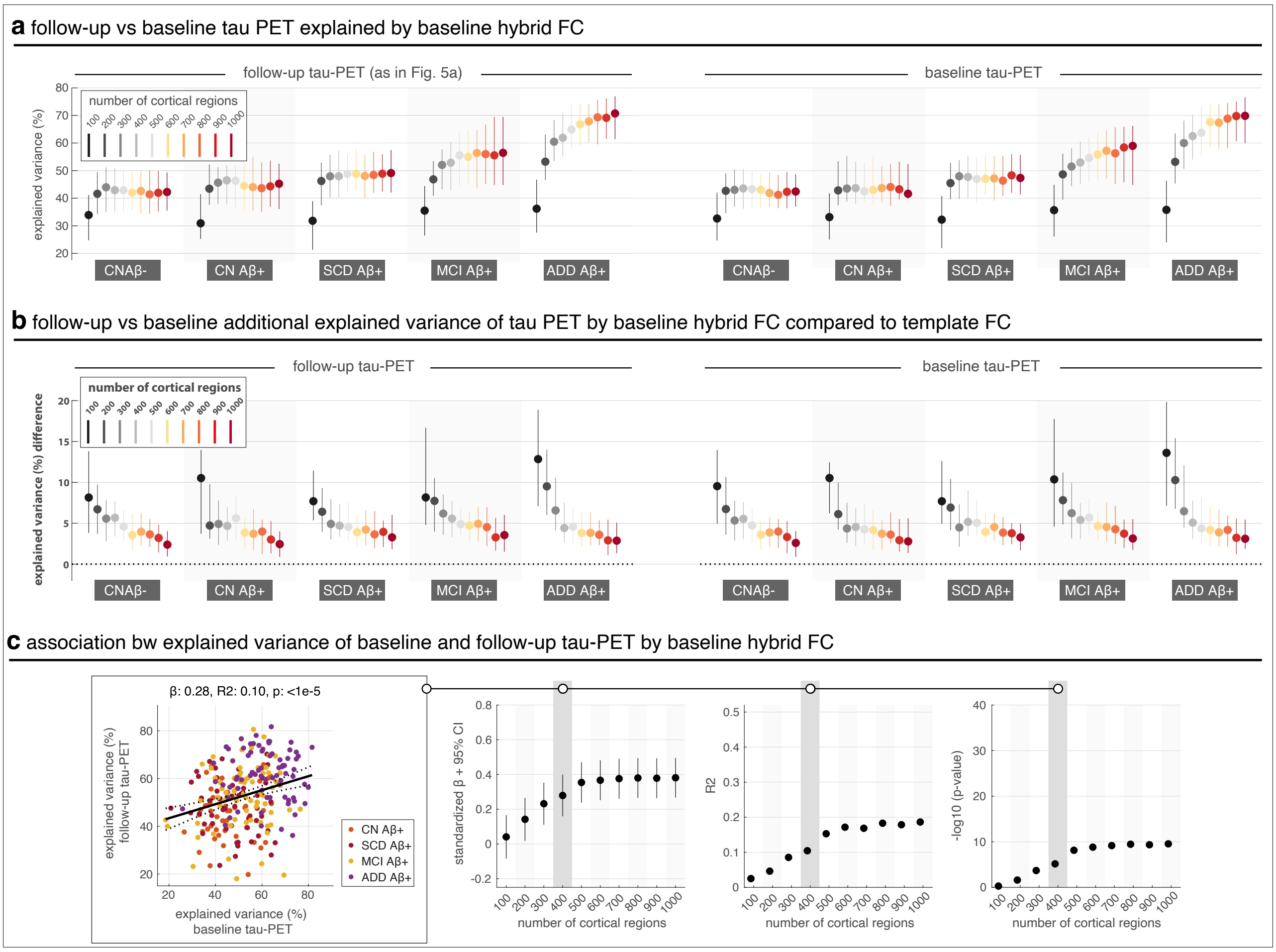

Fig. S12 | Comparison of explained variance in baseline vs. follow-up tau-PET by hybrid FC. (a) Baseline tau-PET patterns were explained by hybrid FC with a similar degree of accuracy as follow-up tau-PET, showing consistent increases with finer parcellations across all clinical groups. (b) Hybrid FC provided added predictive value beyond template FC, especially at finer spatial scales and in MCI Aβ+ and ADD groups. (c) Although group-level variance explained was comparable between baseline and follow-up, individual-level associations between explained variance at the two timepoints were modest (left), with standardized effect sizes peaking around 400–600 cortical regions but with little of the overall variance explained. Together, these results suggest group-level stability in FC-tau alignment, but also underscore subject-specific variability in how tau pathology evolves over time relative to baseline FC architecture.

bioRxiv preprint doi: https://doi.org/10.1101/2025.10.02.679969; this version posted October 14, 2025. The copyright holder for this preprint (which was not certified by peer review) is the author/funder, who has granted bioRxiv a license to display the preprint in perpetuity. It is made available under aCC-BY 4.0 International license.

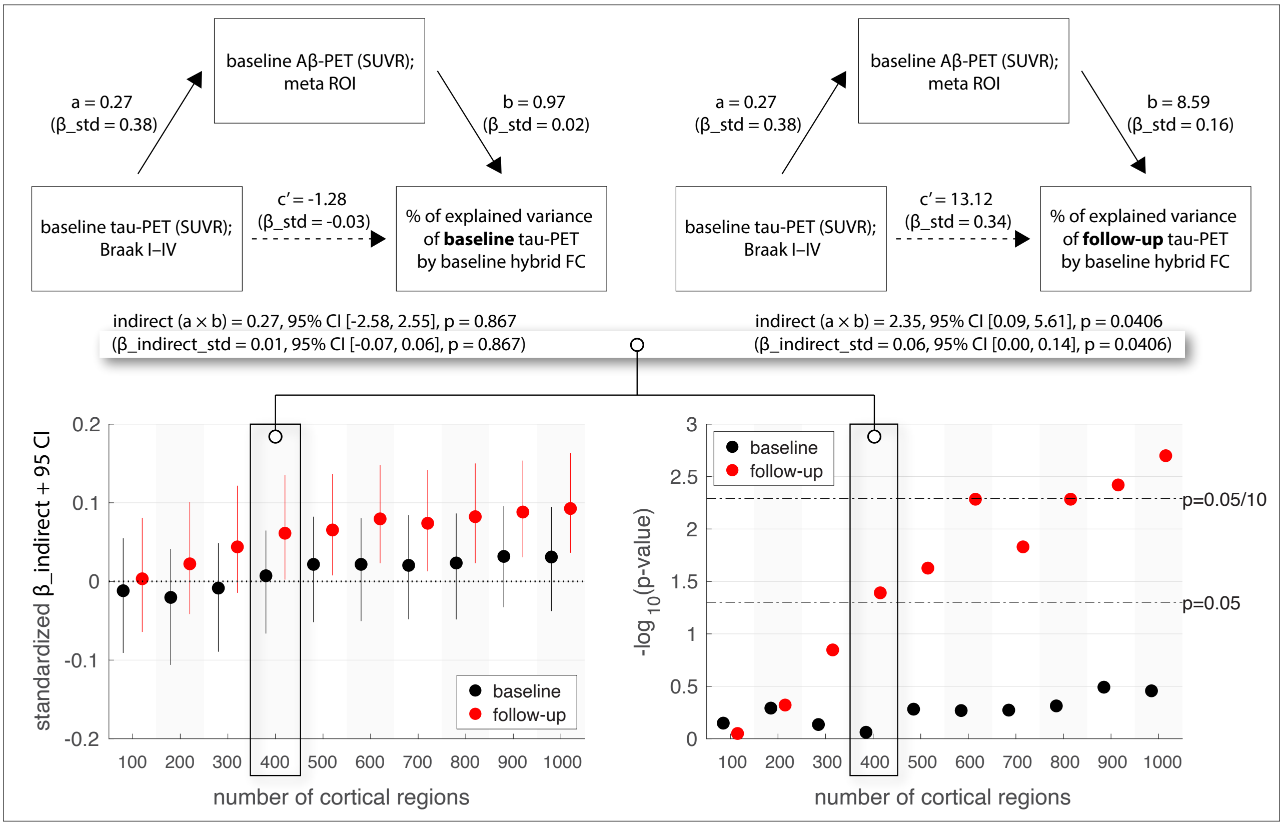

Fig. S13 | Mediation of baseline Aβ-PET between early aggregate tau burden and explained variance (EV) of tau-PET patterns by FC (models adjust for age and sex). Top: Mediation at a 400-region parcellation (unstandardized coefficients; standardized β in parentheses). Aβ significantly mediates the association between baseline Braak I–IV tau and EV follow-up tau-PET patterns predicted by baseline hybrid FC, but not EV of baseline tau-PET patterns. Bottom: Across spatial scales, the mediated effect of Aβ for EV of follow-up tau-PET patterns strengthens with granularity and becomes significant from 400 regions onward, whereas EV of baseline tau-PET patterns shows no mediation at any scale. Same longitudinal sample as used in Fig. 5, where patients with ADD are excluded as they lacked Aβ-PET scans. For Aβ-PET, the meta ROI SUVR is the average SUVR calculated from a global neocortical ROI, including prefrontal, lateral temporal, parietal, anterior cingulate and posterior cingulate/precuneus.
